# Comparative cardiometabolic safety and effectiveness of aripiprazole in people with severe mental illness: A target trial emulation

**DOI:** 10.1371/journal.pmed.1004520

**Published:** 2025-01-23

**Authors:** Alvin Richards-Belle, Naomi Launders, Sarah Hardoon, Al Richards, Kenneth K.C. Man, Neil M. Davies, Elvira Bramon, Joseph F. Hayes, David P.J. Osborn

**Affiliations:** 1 Division of Psychiatry, University College London, London, United Kingdom; 2 Expert by Experience, United Kingdom; 3 Research Department of Practice and Policy, School of Pharmacy, University College London, London, United Kingdom; 4 Centre for Medicines Optimisation Research and Education, University College London Hospitals NHS Foundation Trust, London, United Kingdom; 5 Department of Pharmacology and Pharmacy, Li Ka Shing Faculty of Medicine, University of Hong Kong, Hong Kong; 6 Laboratory of Data Discovery for Health (D24H), Hong Kong Science Park, Pak Shek Kok, Hong Kong; 7 Department of Statistical Sciences, University College London, London, United Kingdom; 8 Department of Public Health and Nursing, Norwegian University of Science and Technology, Trondheim, Norway; 9 Camden and Islington NHS Foundation Trust, London, United Kingdom; Massachusetts General Hospital, UNITED STATES OF AMERICA

## Abstract

**Background:**

There is limited and conflicting evidence on the comparative cardiometabolic safety and effectiveness of aripiprazole in the management of severe mental illness. We investigated the hypothesis that aripiprazole has a favourable cardiometabolic profile, but similar effectiveness when compared to olanzapine, quetiapine, and risperidone.

**Methods and findings:**

We conducted an observational emulation of a head-to-head trial of aripiprazole versus olanzapine, quetiapine, and risperidone in UK primary care using data from the Clinical Practice Research Datalink. We included adults diagnosed with severe mental illness (i.e., bipolar disorder, schizophrenia, and other non-organic psychoses) who were prescribed a new antipsychotic between 2005 and 2017, with a 2-year follow-up to 2019. The primary outcome was total cholesterol at 1 year (cardiometabolic safety). The main secondary outcome was psychiatric hospitalisation (effectiveness). Other outcomes included body weight, blood pressure, all-cause discontinuation, and mortality. Analyses adjusted for baseline confounders, including sociodemographics, diagnoses, concomitant medications, and cardiometabolic parameters.

We included 26,537 patients (aripiprazole, *n* = 3,573, olanzapine, *n* = 8,554, quetiapine, *n* = 8,289, risperidone, *n* = 6,121). Median (IQR) age was 53 (42−67) years, 55.4% were female, 82.3% White, and 18.0% were diagnosed with schizophrenia. Patients prescribed aripiprazole had similar total cholesterol levels after 1 year to those prescribed olanzapine (adjusted mean difference [aMD], −0.03, 95% CI, −0.09 to 0.02, *p* = 0.261), quetiapine (aMD, −0.03, 95% CI, −0.09 to 0.03, *p* = 0.324), and risperidone (aMD, −0.01, 95% CI, −0.08 to 0.05, *p* = 0.707). However, there was evidence that patients prescribed aripiprazole had better outcomes on other cardiometabolic parameters, such as body weight and blood pressure, especially compared to olanzapine. After additional adjustment for prior hospitalisation, patients prescribed aripiprazole had similar rates of psychiatric hospitalisation as those prescribed olanzapine (adjusted hazard ratio [aHR], 0.91, 95% CI, 0.82 to 1.01, *p* = 0.078), quetiapine (aHR, 0.94, 95% CI, 0.85 to 1.04, *p* = 0.230), or risperidone (aHR, 1.01, 95% CI, 0.91 to 1.12, *p* = 0.854).

**Conclusions:**

Data from our large, powered, diverse, real-world target trial emulation sample, followed over 2 years, suggest that adults diagnosed with severe mental illness prescribed aripiprazole have similar total cholesterol 1 year after first prescription compared to those prescribed olanzapine, quetiapine, and risperidone. However, patients prescribed aripiprazole had better outcomes on some other cardiometabolic parameters, and there was little evidence of differences in effectiveness. Our findings inform a common clinical dilemma and contribute to the evidence base for real-world clinical decision-making on antipsychotic choice for patients diagnosed with severe mental illness.

## Author summary

**Why was this study done?:**

- Antipsychotics are the mainstay of treatment in severe mental illness. The decision to prescribe one medication over another requires careful evaluation of potential risks and benefits. Aripiprazole was licensed in the United Kingdom (UK) in 2004, where it has become one of the most frequently prescribed antipsychotics—alongside olanzapine, quetiapine, and risperidone.
- Systematic reviews of randomised evidence typically report aripiprazole as similar or less effective in reducing primary symptoms (psychotic, manic, or depressive symptoms, as appropriate) than olanzapine, quetiapine, and risperidone, but similar or favourable on cardiometabolic safety parameters (such as total cholesterol and body weight). However, there are methodological concerns about randomised trials of antipsychotic and, in particular, their generalisability to real-world practice.
- Reviews of real-world evidence have highlighted aripiprazole as among the least metabolically characterised second-generation antipsychotics, and no comparative real-world cardiometabolic safety data has come from UK practice.

**What did the researchers do and find?:**

- We emulated a head-to-head trial of aripiprazole versus olanzapine, quetiapine, and risperidone for patients diagnosed with severe mental illness in UK primary care and provide real-world evidence on comparative cardiometabolic safety and psychiatric effectiveness.
- In this target trial emulation involving 26,537 patients, there was little evidence of differences in total cholesterol levels after 1 year or in the rate of psychiatric hospitalisation among patients prescribed aripiprazole when compared to those prescribed olanzapine, quetiapine, or risperidone. However, patients prescribed aripiprazole had better outcomes on other cardiometabolic parameters, such as body weight and blood pressure, especially when compared to patients prescribed olanzapine.

**What do these findings mean?:**

- Data from our large, powered, diverse, real-world target trial emulation, which followed patients over 2 years, suggest that aripiprazole might confer some comparative cardiometabolic benefits over the comparators without compromising psychiatric effectiveness.
- Our findings inform a common clinical dilemma and contribute to the evidence base for real-world clinical decision-making on antipsychotic choice for patients diagnosed with severe mental illness.
- Although our analyses accounted for many measured potential confounders, there is a risk of residual and unmeasured confounding as we could not account for factors such as functional impairment and psychiatric service contact. There was missing data for cardiometabolic safety outcomes—although we used multiple imputation to handle missing data, our results may not generalise to people who engage less, or not at all, with primary care.

## Abstract 3

In a target trial emulation, Alvin Richards-Belle and colleagues compare the effectiveness and safety profile of aripiprazole and other second-generation antipsychotics in patients diagnosed with severe mental illness in UK primary care.

## Introduction

Severe mental illnesses (SMIs), such as schizophrenia and bipolar disorder, are associated with substantial illness burden for patients, including premature mortality [1]. Antipsychotics are the mainstay of treatment, and, although effective at reducing symptoms for many [2], their use is not without risk. Adverse effects include dyslipidaemia, insulin resistance, and weight gain [3,4], which increase the risk for cardiovascular diseases [5] and contribute to premature mortality [1]. The decision to prescribe one medication over another, therefore, requires careful evaluation of potential risks and benefits.

Aripiprazole, a dopamine and serotonin receptor partial agonist, was licensed in the United Kingdom (UK) in 2004 [6], where it is now one of the most frequently prescribed antipsychotics—alongside olanzapine, quetiapine, and risperidone [7]. Compared to the latter three agents, syntheses of randomised clinical trial (RCT) evidence typically report aripiprazole as similar or less effective in reducing primary symptoms [2,8–12], but similar or favourable on cardiometabolic safety parameters [2–4,8–11]. However, although RCTs are the gold standard for estimating causal effects, there are concerns about existing RCTs of antipsychotics—especially their generalisability to real-world practice [13]. Such RCTs have often been limited by small, non-representative samples [14], short follow-ups, poor retention, and limited safety reporting [15]—with many rated as at high or unclear risk of bias [2,4,9–11]. New RCTs designed to overcome these methodological limitations would be ideal for advancing the evidence base—but RCTs are time-consuming, challenging, and expensive to conduct—and therefore not practical to study effects which may emerge after years of exposure.

Observational studies using electronic health records are increasingly recognised as complementary to RCTs, providing real-world evidence and being well-suited for evaluating long-term outcomes [16]. However, to date, only one study has evaluated aripiprazole in UK practice. Montastruc and colleagues investigated aripiprazole as a switch from or add-on to a previous antipsychotic, compared with any other antipsychotic used in the same manner, and found little evidence of differences in risks of psychiatric hospitalisation, self-harm, or suicide [17]. Since the comparator was any other antipsychotic, results cannot be generalised to individual antipsychotic comparisons. More broadly, reviews of real-world evidence highlight aripiprazole as among the least metabolically characterised second-generation antipsychotics [18], with no comparative cardiometabolic data from UK practice [18–20].

Adopting the target trial emulation framework [21], a causal inference approach that aims to avoid methodological flaws and minimise bias in observational studies by emulating the key design features of RCTs, we sought to emulate a pragmatic RCT on the following question: In adults diagnosed with SMI who are prescribed a new antipsychotic in primary care, does aripiprazole, as compared to olanzapine, quetiapine, and risperidone, have a better cardiometabolic safety profile with similar comparative effectiveness?

## Methods

### Study design

We conducted an observational, electronic health record, head-to-head, pragmatic, target trial emulation study. We designed the protocol for a hypothetical target trial, which specified that, following a clinical decision to initiate aripiprazole, olanzapine, quetiapine, or risperidone in primary care, eligible patients would be randomised to initiate monotherapy with one of the four antipsychotics and followed over 2 years for outcomes. We mapped the key design features of the *target trial* to the design of this *emulation* (Table 1). The study protocol was pre-registered before the review of outcome data [22] (S1 Protocol) and the study is reported according to Strengthening the Reporting of Observational Studies in Epidemiology (STROBE) guidelines (S1 STROBE Checklist).

**Table 1:** Key design features of a hypothetical ideal target trial and the target trial emulation comparing aripiprazole with olanzapine, quetiapine, and risperidone^*^.

Trial design	Hypothetical ideal target trial	Target trial emulation
Patient inclusion criteria	Clinical decision to initiate aripiprazole, olanzapine, quetiapine, or risperidone as oral monotherapy in primary care.	First-time prescription of aripiprazole, olanzapine, quetiapine, or risperidone as oral monotherapy in primary care.
	Age 18–99 years.	Age 18–99 years.
	Severe mental illness diagnosis.	Severe mental illness diagnosis recorded in primary care.
	Baseline blood test for lipids and HbA1c values.	Value(s) for lipids or HbA1c recorded at least once in primary care in the prior 2y.
		Registered at primary care practice for at least 6m.
Patient exclusion criteria	Dementia diagnosis.	Dementia diagnosis recorded in primary care.
	Prescription of more than one antipsychotic.	Prescription of more than one antipsychotic in primary care.
	Prescription of a long-acting injectable ‘depot’ antipsychotic in the last 90 days.	Prescription of a long-acting injectable ‘depot’ antipsychotic in primary care in the last 90 days.
Recruitment period	2005–2017	2005–2017
Follow-up duration	Each patient followed-up to a maximum of 2y following randomisation date.	Each patient followed-up to a maximum of 2y following index date, defined as the date on which eligibility criteria were met and treatment was assigned.
Outcome(s)	Primary outcome: Total cholesterol at 1y	Primary outcome: Total cholesterol at 1y
	Main secondary outcome: Psychiatric hospitalisation censored at 2y	Main secondary outcome: Psychiatric hospitalisation censored at 2y^a^
	Secondary cardiometabolic safety outcomes: Total cholesterol, LDL-C, HDL-C, triglycerides, TC:HDL ratio, body weight, systolic blood pressure, diastolic blood pressure, HbA1c, glucose—all at 6m, 1y and 2y	Secondary cardiometabolic safety outcomes: Total cholesterol, LDL-C, HDL-C, triglycerides, TC:HDL ratio, body weight, systolic blood pressure, diastolic blood pressure, HbA1c, glucose—all at 6m, 1y and 2y
	Secondary effectiveness outcomes: All-cause discontinuation, all-cause mortality—censored at 2y	Secondary effectiveness outcomes: All-cause discontinuation, all-cause mortality—censored at 2y
Treatments strategies	Oral monotherapy with either: Aripiprazole (intervention) Olanzapine Quetiapine Risperidone	Prescription of either: Aripiprazole (main exposure) Olanzapine Quetiapine Risperidone
Assignment procedures	Randomisation	Patients assigned to the treatment strategy with which their data are consistent with. Baseline covariate adjustment used in an attempt to conditionally emulate randomisation. Candidate confounders selected based on clinical expertise and prior research guided by a directed acyclic graph [23].
Causal contrasts of interest	Intention-to-treat (primary) Per-protocol (supplementary)	Observational analogues of: Intention-to-treat (primary) Per-protocol (supplementary)
Estimand	Average treatment effect	Average treatment effect
Analysis plan	Cardiometabolic outcomes (including the primary outcome): Linear regression to estimate the adjusted mean difference for aripiprazole vs. each comparator.	Cardiometabolic outcomes (including the primary outcome): Linear regression to estimate the adjusted mean difference for aripiprazole vs. each comparator.
	Effectiveness outcomes: Cox regression to estimate the adjusted hazard ratio for aripiprazole vs. each comparator.	Effectiveness outcomes: Cox regression to estimate the adjusted hazard ratio for aripiprazole vs. each comparator.

*Table adapted from Hernan and Robins (2016) [21].

^a^Available in a subset of patients with linked Hospital Episode Statistics (HES) data.

LDL-C, low-density lipoprotein cholesterol; HDL-C, high-density lipoprotein cholesterol; TC:HDL, total cholesterol to high-density lipoprotein; HbA1c, glycated haemoglobin.

### Lived experience involvement

Two lived experience advisors were recruited to the study advisory committee. They were involved in the study design, oversight, interpretation, and dissemination of the results and they are included in this article either as a co-author or are acknowledged for their contribution. The advisors brought invaluable expertise on the real-world effects of antipsychotic medications. They helped focus the study on those effects that most impact quality of life (including through prioritising cardiometabolic outcomes—which informed the selection of the primary outcome).

### Data source

We used de-identified data from Clinical Practice Research Datalink (CPRD) GOLD [24] and Aurum [25] databases, which contain the primary care records (including diagnoses, prescriptions, and test results) of over 62 million (current and historic) patients and are broadly representative of the UK population. We used the April 2023 and May 2022 builds of GOLD and Aurum, respectively. We leveraged linkage to Hospital Episode Statistics (HES) [26] and the Office for National Statistics (ONS) for data on hospital admissions and registered deaths in England. Ethical approval was obtained from the Independent Scientific Advisory Committee of CPRD (protocol no. 21_000729).

### Population

Eligible patients were those identified as meeting eligibility as at the date of first prescription of a study antipsychotic in primary care (index date). Inclusion criteria were SMI diagnosis (i.e., schizophrenia, bipolar disorder, and other non-organic psychoses) recorded in primary care, age 18–99 years, primary care registration for at least 6 months, and lipids or glycated haemoglobin (HbA1c) recorded at least once in the prior 2 years. Exclusion criteria were dementia diagnosis, prescription of more than one antipsychotic, and prescription of a long-acting injectable antipsychotic in the prior 90 days.

Allowing for recording delays, SMI diagnosis could be recorded up to 30 days after the index date, given such diagnoses are typically made by psychiatrists in secondary care and subsequently communicated to primary care. ‘Other non-organic psychoses’ included non-affective psychoses such as delusional disorders, schizoaffective disorders, and non-organic psychosis not otherwise specified. Our clinician-verified code list for all SMI diagnoses is publicly available [27]. The validity of SMI diagnoses in primary care has been established [28].

Patients fulfilling eligibility from 1 January 2005–31 December 2017 entered the study on the index date. They exited at the earliest of 2-year follow-up (final follow-up, 31 December 2019), de-registration, death, or administrative censoring. Patients could only meet eligibility criteria once (i.e., at the first prescription).

### Exposures

The first prescription of aripiprazole in primary care was considered the intervention and the first prescription of either olanzapine, quetiapine, or risperidone as the comparators. Antipsychotic prescriptions, identified using relevant product codes [7], could be initiated by general practitioners or specialists (e.g., psychiatrists), but must have been issued and recorded in primary care (standard practice for community management in the UK).

### Outcomes

#### Primary outcome.

In consultation with lived experience advisors, we focussed the primary outcome on cardiometabolic safety. We chose total cholesterol at 1 year as the primary outcome given it is a well-established risk factor for cardiovascular morbidity, lipids are understudied in real-world evidence for aripiprazole [18,19], and RCTs in this population used this primary outcome [29].

#### Main secondary outcome.

The main secondary outcome focussed on comparative effectiveness: psychiatric hospitalisation, defined using HES data as a hospitalisation record in which, during an episode of care, a psychiatric condition was coded in the primary diagnosis position (or intentional self-harm in any position) (Table A in S1 Appendix).

#### Other secondary outcomes.

Other cardiometabolic safety outcomes were: low-density lipoprotein cholesterol (LDL-C); high-density lipoprotein cholesterol (HDL-C); triglycerides; total cholesterol to HDL-CDL) ratio; body weight; systolic blood pressure; diastolic blood pressure; glucose; and HbA1c—all at 6 months, 1 year, and 2 years. Allowing for varying measurement times, we widened windows for the recording of cardiometabolic outcomes (e.g., 6 ± 3 months, 12 ± 3 months, and 24 ± 6 months), and used values closest to the time point. Recorded values were considered valid if within reference ranges (Table B in S1 Appendix).

Other effectiveness outcomes were all-cause discontinuation and mortality. Patients were considered to have discontinued the study antipsychotic if there was a period of ≥ 90 days where the antipsychotic was not prescribed. Mortality was defined using CPRD and ONS data [30].

### Covariates

Outcome models were adjusted for the following pre-specified baseline covariates: age, sex, ethnicity, SMI diagnosis, prior use of non-study antipsychotics, relative deprivation, geographic region, index year, number of primary care consultations in prior 6 months, smoking status, comorbidities (alcohol misuse, cerebrovascular disease, diabetes, dyslipidaemia, hypertension, liver disease, myocardial infarction, renal disease, substance misuse), concomitant medications (antidepressants, mood stabilisers, lipid-regulating medications, antidiabetics, antihypertensives), body mass index category, and cardiometabolic values (see Table C in S1 Appendix for details). These covariates were selected as candidate confounders based on clinical expertise and prior research, guided by a directed acyclic graph (Fig A in S1 Appendix) [23]. Variables were defined according to codes recorded in CPRD. Where possible, existing code lists were used [27,31]. Otherwise, they were generated using keyword search strategies applied to code dictionaries.

### Missing data

Assuming data were missing at random, missing covariates and cardiometabolic outcomes were imputed using multiple imputation by chained equations, implemented in the *mice* R package [32]. Predictive mean matching was used for continuous variables and multinomial logistic regression was used for categorical variables. Twenty-five datasets were generated using an imputation model comprising baseline, auxiliary, and outcome variables (see Table D in S1 Appendix for details). Patterns of missingness were reviewed to assess the plausibility of the missing at random assumption.

### Statistical analysis

Primary analyses followed the intention-to-treat (observational analogue) principle, with patients analysed according to their baseline antipsychotic group, irrespective of adherence or discontinuation.

For cardiometabolic outcomes, we used linear regression to estimate adjusted mean differences (aMDs) for aripiprazole versus each comparator antipsychotic at each time point. For effectiveness outcomes, we used Cox regression to estimate adjusted hazard ratios (aHRs) for aripiprazole versus each comparator, with follow-up censored at 2 years. Since treatment effects might vary over time, these HRs should be interpreted as weighted averages of the instantaneous risks over follow-up. Hospitalisation and discontinuation were additionally analysed accounting for death as a potential competing risk using Fine-Gray regression. Models were fitted in each imputed dataset, with coefficients pooled using Rubin’s rules. Patients without linked HES data were excluded from the hospitalisation analysis (this outcome was not imputed).

We explored whether results for total cholesterol at 1 year and psychiatric hospitalisation (censored at 2 years) varied by age, sex, ethnicity, diagnosis, prior use of non-study antipsychotics, and time-periods. For each characteristic, we compared confounder-adjusted models with and without interaction terms using analysis of variance (ANOVA) for total cholesterol and Wald tests for hospitalisation; *p* < 0.05 was used to determine the presence of effect modification.

Statistical tests used to analyse outcomes were two-sided, used a 5% significance level, and estimates are reported with 95% confidence intervals (CIs). *P*-values were not adjusted for multiple comparisons as we pre-specified a primary outcome and we report *P*-values for the main outcomes and effect modification tests only. A pre-specified illustrative power calculation for the primary outcome is shown in Table E in S1 Appendix. All analyses were conducted in R (version 4.4.0) [33].

#### Supplementary and sensitivity analyses.

In a per-protocol supplementary analysis, analyses for all outcomes were repeated, with patients censored at initiation of another study antipsychotic, so that outcomes could be attributed to a particular antipsychotic, or at discontinuation date, to allow for potential later development of adverse effects and delayed reporting.

In a weighted sensitivity analysis, analyses for total cholesterol at 1 year and psychiatric hospitalisation (censored at 2 years) were repeated using inverse probability of treatment weighting as an alternative approach to reduce confounding bias. Inverse probability of treatment weighting creates a pseudo-population based on the propensity score, weighted by the inverse of the probability of receiving the treatment that was received. Propensity scores were estimated using multinomial logistic regression to predict the prescription of each of the four antipsychotics, using all covariates mentioned previously. Stabilised weights were used to reduce the impact of potential extreme weights. We compared standardised mean differences before and after weighting to observe the success of covariate balance, using a threshold for difference of > 0.1 to indicate potentially meaningful imbalance [34]. We planned to consider interactions and other non-linear forms to iteratively improve propensity score specifically and to use doubly robust methods if imbalances remained.

In post hoc analyses, total cholesterol at 1 year and psychiatric hospitalisation were analysed with robust standard errors accounting for clustering by general practice and the Cox regression model for psychiatric hospitalisation was additionally adjusted for prior psychiatric hospitalisation due to baseline imbalance. In response to reviewer comments, per-protocol analyses for the main outcomes were also conducted with inverse probability of censoring weighting to account for potential differential censoring across treatment groups. Stabilised censoring weights were derived from a logistic model incorporating available baseline covariates and follow-up variables (i.e., indicators of missing data and cardiometabolic values over follow-up time points), with trimming at the 99.5th percentile.

#### Role of funding source.

The funding sources had no role in study design, in the collection, analysis, and interpretation of data, in the writing of the report, or in the decision to submit the paper for publication.

## Results

**Fig 1:**
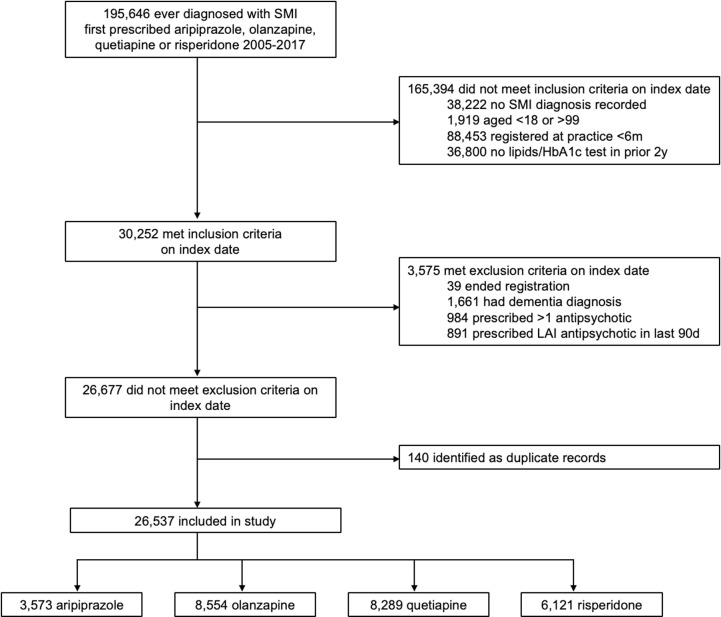
Study flow diagram. SMI, severe mental illness; HbA1c, glycated haemoglobin; LAI, long-acting injectable.

Among the 195,646 patients identified as first prescribed a study antipsychotic in primary care between 2005 and 2017, 26,537 met eligibility and were included (aripiprazole, *n* = 3,573; olanzapine, *n* = 8,554; quetiapine, *n* = 8,289; risperidone, *n* = 6,121) (Fig 1). Included patients were older, more likely to be female, and less likely to have been diagnosed with schizophrenia than patients not meeting eligibility, but there were limited differences according to ethnicity, region, and deprivation (Table F in S1 Appendix).

Baseline characteristics of the treatment groups before imputation and adjustment are shown in Table 2. Patients in the aripiprazole group had the lowest median age (49 years), the most ethnic diversity (24.0% ethnic minorities), and the highest proportion diagnosed with schizophrenia (29.9%). The quetiapine group had the highest proportions of females (60.1%), those diagnosed with bipolar disorder (58.9%), and of White ethnicity (88.6%). The risperidone group had the highest median age (57 years) and proportion diagnosed with other non-organic psychoses (i.e., not schizophrenia or bipolar disorder) (54.5%). There was no clear demographic pattern for patients prescribed olanzapine, but these patients had the lowest mean body mass index (26.9 kg/m^2^) and proportion diagnosed with diabetes (14.3%) at baseline. The mean (SD) total cholesterol of patients prescribed aripiprazole at baseline was 4.94 (1.19) mmol/L, which compared to 5.10 (1.18), 5.07 (1.17), and 4.95 (1.16) in those prescribed olanzapine, quetiapine, and risperidone, respectively. Over a quarter (27.5%) of patients prescribed aripiprazole had a psychiatric hospitalisation in the 2 years before baseline, which compared to 34.9%, 23.7%, and 25.7% in those prescribed olanzapine, quetiapine, and risperidone, respectively.

**Table 2:** Baseline characteristics.

Characteristic	Aripiprazole *N* = 3,573	Olanzapine *N* = 8,554	Quetiapine *N* = 8,289	Risperidone *N* = 6,121
**Age at baseline (years), median (IQR)**	49 (38, 62)	54 (42, 67)	52 (41, 66)	57 (45, 72)
**Sex, No. (%)**				
Female	1,970 (55.1%)	4,419 (51.7%)	4,982 (60.1%)	3,339 (54.5%)
Male	1,603 (44.9%)	4,135 (48.3%)	3,307 (39.9%)	2,782 (45.5%)
**Ethnicity, No. (%)**				
Asian	331 (10.2%)	574 (7.5%)	393 (5.4%)	509 (9.2%)
Black	324 (10.0%)	518 (6.7%)	241 (3.3%)	566 (10.2%)
Mixed	73 (2.3%)	114 (1.5%)	99 (1.4%)	101 (1.8%)
Other	48 (1.5%)	114 (1.5%)	99 (1.4%)	95 (1.7%)
White	2,466 (76.1%)	6,376 (82.8%)	6,493 (88.6%)	4,265 (77.0%)
Unknown	331	858	964	585
**Diagnosis, No. (%)**				
Bipolar disorder	1,144 (32.0%)	3,521 (41.2%)	4,879 (58.9%)	1,434 (23.4%)
Other non-organic psychoses	1,361 (38.1%)	3,553 (41.5%)	2,522 (30.4%)	3,339 (54.5%)
Schizophrenia	1,068 (29.9%)	1,480 (17.3%)	888 (10.7%)	1,348 (22.0%)
**Age at diagnosis (years), median (IQR)**	38 (27, 52)	44 (31, 58)	43 (31, 56)	47 (33, 65)
**Years from first diagnosis to index date, median (IQR)**	3.8 (0.2, 14.7)	1.9 (0.1, 14.1)	2.9 (0.1, 12.6)	1.3 (0.1, 13.4)
**Prescribed a non**-**study antipsychotic in last 2y**^a^**, No. (%)**	911 (25.5%)	1,679 (19.6%)	1,628 (19.6%)	1,405 (23.0%)
**Psychiatric hospitalisation in last 2y, No. (%)**	802 (27.5%)	2,423 (34.9%)	1,543 (23.7%)	1,303 (25.7%)
Unknown	655	1,605	1,786	1,051
**Index date time-period, No. (%)**				
2005–2009	619 (17.3%)	3,078 (36.0%)	2,387 (28.8%)	2,027 (33.1%)
2010–2014	1,547 (43.3%)	3,390 (39.6%)	3,828 (46.2%)	2,742 (44.8%)
2015+	1,407 (39.4%)	2,086 (24.4%)	2,074 (25.0%)	1,352 (22.1%)
**IMD quintile, No. (%)**				
1 (Least deprived)	344 (11.5%)	1,000 (14.1%)	1,012 (15.2%)	607 (11.7%)
2	440 (14.8%)	1,110 (15.6%)	1,140 (17.1%)	792 (15.3%)
3	542 (18.2%)	1,324 (18.6%)	1,300 (19.6%)	987 (19.1%)
4	765 (25.7%)	1,678 (23.6%)	1,403 (21.1%)	1,285 (24.8%)
5 (Most deprived)	890 (29.9%)	1,992 (28.0%)	1,793 (27.0%)	1,508 (29.1%)
Unknown	592	1,450	1,641	942
**Comorbidities, No. (%)**				
Alcohol misuse	368 (10.3%)	950 (11.1%)	991 (12.0%)	575 (9.4%)
Cerebrovascular disease	211 (5.9%)	508 (5.9%)	570 (6.9%)	512 (8.4%)
Diabetes	990 (27.7%)	1,220 (14.3%)	1,657 (20.0%)	1,580 (25.8%)
Dyslipidaemia	729 (20.4%)	1,614 (18.9%)	1,672 (20.2%)	1,424 (23.3%)
Hypertension	1,012 (28.3%)	2,451 (28.7%)	2,436 (29.4%)	2,264 (37.0%)
Liver disease	88 (2.5%)	211 (2.5%)	172 (2.1%)	116 (1.9%)
Myocardial infarction	118 (3.3%)	270 (3.2%)	263 (3.2%)	285 (4.7%)
Renal disease	355 (9.9%)	838 (9.8%)	907 (10.9%)	809 (13.2%)
Substance misuse	338 (9.5%)	743 (8.7%)	641 (7.7%)	373 (6.1%)
**Concomitant medications, No. (%)**				
Antidepressant^b^	1,988 (55.6%)	4,862 (56.8%)	5,791 (69.9%)	3,174 (51.9%)
Mood stabiliser^c^	879 (24.6%)	2,496 (29.2%)	3,185 (38.4%)	1,126 (18.4%)
Lipid-regulating medication^d^	1,158 (32.4%)	2,262 (26.4%)	2,540 (30.6%)	2,242 (36.6%)
Antidiabetic^e^	682 (19.1%)	612 (7.2%)	1,042 (12.6%)	1,059 (17.3%)
Antihypertensive^f^	1,325 (37.1%)	3,193 (37.3%)	3,393 (40.9%)	2,768 (45.2%)
**Total cholesterol (mmol/L), mean (SD)** ^g^	4.94 (1.19)	5.10 (1.18)	5.07 (1.17)	4.95 (1.16)
Unknown	240	480	512	279
**BMI (mg/m** ^**2**^ **), mean (SD)**	29.9 (7.5)	26.9 (5.7)	28.5 (6.5)	28.3 (6.5)
Unknown	641	2,109	1,721	1,265
**BMI category, No. (%)**				
Underweight	73 (2.5%)	262 (4.1%)	167 (2.5%)	145 (3.0%)
Healthy	754 (25.7%)	2,327 (36.0%)	1,925 (29.2%)	1,502 (30.8%)
Overweight	843 (28.7%)	2,217 (34.3%)	2,186 (33.2%)	1,539 (31.6%)
Obese	1,267 (43.1%)	1,650 (25.6%)	2,309 (35.1%)	1,685 (34.6%)
Unknown	636	2,098	1,702	1,250
**Smoking status, No. (%)**				
Never smoked	1,402 (39.4%)	3,329 (39.2%)	3,268 (39.6%)	2,590 (42.5%)
Ex-smoker	614 (17.2%)	1,361 (16.0%)	1,468 (17.8%)	1,031 (16.9%)
Current smoker	1,544 (43.4%)	3,810 (44.8%)	3,526 (42.7%)	2,479 (40.6%)
Unknown	13	54	27	21
**Starting daily dose (Olanzapine equivalent, mg), mean (SD)** ^h^	6.7 (3.9)	8.5 (5.7)	4.0 (4.4)	4.1 (3.4)
Unknown	576	1,266	1,897	1,079

Data are shown before imputation and adjustment.

Concomitant medications defined according to prescriptions on or within the 2 years prior to the index date. Comorbidities determined on or prior to the index date, using the patient’s full available medical history.

^a^The most frequent previously prescribed non-study antipsychotics were chlorpromazine, trifluoperazine, haloperidol, and amisulpride.

^b^The most frequently concomitantly prescribed antidepressants were citalopram, mirtazapine, sertraline, amitriptyline, fluoxetine, and venlafaxine.

^c^The most frequently concomitantly prescribed mood stabilisers were lithium and sodium valproate.

^d^The most frequently concomitantly prescribed lipid-regulating medications were simvastatin and atorvastatin.

^e^The most frequently concomitantly prescribed antidiabetics were metformin, gliclazide, and insulin.

^f^The most frequently concomitantly prescribed antihypertensives were amlodipine, ramipril, and bendroflumethiazide.

^g^To convert from mmol/L to mg/dL, multiply by 38.67.

^h^Calculated according to the Defined Daily Dose method [35] and expressed as an olanzapine equivalent dose.

IMD, index of multiple deprivation; BMI, body mass index; mg, milligrams.

Unadjusted cardiometabolic parameters are shown in Table G in S1 Appendix, with levels of missing data in Table H in S1 Appendix. The missing at random assumption was considered plausible for the primary outcome (Table I in S1 Appendix).

Across imputed datasets and following inverse probability of treatment weighting, mean standardised differences for all covariates between the aripiprazole and each comparator group were < 0.1, demonstrating covariate balance (Fig B in S1 Appendix). The distribution of weights overlapped, with mean weights close to 1.0 across comparison groups (Fig C in S1 Appendix).

### Cardiometabolic safety

#### Primary outcome.

After confounder adjustment, patients prescribed aripiprazole had similar total cholesterol 1 year after first prescription as those prescribed olanzapine (aMD, −0.03, 95% CI, −0.09 to 0.02, *p* = 0.261), quetiapine (aMD, −0.03, 95% CI, −0.09 to 0.03, *p* = 0.324), and risperidone (aMD, −0.01, 95% CI, −0.08 to 0.05, *p* = 0.707) (Table 3). Estimates from per-protocol (with and without censoring weighting) (Table J in S1 Appendix), inverse probability of treatment weighting, and primary care practice clustering (Table K in S1 Appendix) analyses were consistent with these findings. We found little evidence of heterogeneity across subgroups (Fig D in S1 Appendix). Patients prescribed aripiprazole had similar levels of prescriptions for lipid-regulating medications during follow-up as the comparators (Table L in S1 Appendix).

**Table 3:** Cardiometabolic safety outcomes with aripiprazole vs. comparator antipsychotics^*^.

	Aripiprazole vs. Olanzapine	Aripiprazole vs. Quetiapine	Aripiprazole vs. Risperidone
**Total cholesterol (mmol/L)**			
6m	−0.12 (−0.19, −0.04)	−0.04 (−0.11, 0.02)	−0.03 (−0.12, 0.06)
1y	−0.03 (−0.09, 0.02)	−0.03 (−0.09, 0.03)	−0.01 (−0.08, 0.05)
2y	0.01 (−0.05, 0.06)	0.01 (−0.04, 0.06)	0.03 (−0.03, 0.09)
**LDL-C (mmol/L)**			
6m	−0.08 (−0.16, −0.01)	−0.01 (−0.08, 0.06)	−0.02 (−0.11, 0.07)
1y	−0.04 (−0.11, 0.03)	−0.03 (−0.10, 0.04)	−0.02 (−0.10, 0.05)
2y	−0.04 (−0.10, 0.02)	−0.02 (−0.07, 0.03)	−0.02 (−0.08, 0.04)
**HDL-C (mmol/L)**			
6m	0.02 (−0.01, 0.06)	0.02 (−0.01, 0.05)	0.01 (−0.02, 0.04)
1y	0.03 (0.01, 0.05)	0.01 (−0.01, 0.03)	0.02 (−0.01, 0.04)
2y	0.03 (0.01, 0.05)	0.02 (0.00, 0.04)	0.03 (0.01, 0.05)
**Triglycerides (mmol/L)**			
6m	−0.14 (−0.23, −0.05)	−0.12 (−0.20, −0.04)	−0.06 (−0.16, 0.04)
1y	−0.07 (−0.14, 0.01)	−0.04 (−0.11, 0.03)	−0.01 (−0.09, 0.07)
2y	0.02 (−0.06, 0.09)	0.01 (−0.06, 0.09)	0.05 (−0.04, 0.14)
**TC:HDL ratio**			
6m	−0.16 (−0.25, −0.07)	−0.06 (−0.15, 0.03)	−0.05 (−0.15, 0.04)
1y	−0.11 (−0.18, −0.04)	−0.04 (−0.11, 0.03)	−0.06 (−0.15, 0.02)
2y	−0.07 (−0.14, 0.00)	−0.03 (−0.10, 0.04)	−0.06 (−0.13, 0.01)
**Weight (kg)**			
6m	−1.03 (−1.52, −0.54)	0.10 (−0.46, 0.66)	−0.25 (−0.87, 0.37)
1y	−1.50 (−2.10, −0.91)	−0.41 (−0.98, 0.16)	−0.76 (−1.41, −0.11)
2y	−0.39 (−1.10, 0.32)	0.54 (−0.20, 1.28)	0.06 (−0.64, 0.76)
**Systolic blood pressure (mmHg)**			
6m	−0.90 (−1.83, 0.04)	−0.86 (−1.86, 0.14)	−0.30 (−1.20, 0.61)
1y	−1.04 (−2.01, −0.08)	−1.14 (−2.06, −0.22)	−0.44 (−1.55, 0.68)
2y	−0.28 (−1.23, 0.67)	−0.66 (−1.48, 0.17)	0.42 (−0.47, 1.32)
**Diastolic blood pressure (mmHg)**			
6m	−1.10 (−1.67, −0.54)	−0.55 (−1.16, 0.05)	−0.33 (−0.88, 0.21)
1y	−0.92 (−1.46, −0.38)	−0.80 (−1.39, −0.21)	−0.05 (−0.76, 0.65)
2y	−0.78 (−1.37, −0.20)	−0.68 (−1.27, −0.08)	−0.21 (−0.79, 0.36)
**HbA1c (mmol/mol)**			
6m	−1.16 (−2.07, −0.25)	−0.48 (−1.36, 0.39)	−0.71 (−1.56, 0.13)
1y	−0.20 (−1.15, 0.75)	−0.11 (−0.92, 0.71)	0.07 (−0.75, 0.89)
2y	0.40 (−0.26, 1.05)	−0.03 (−0.74, 0.67)	0.64 (−0.04, 1.32)
**Glucose (mmol/L)**			
6m	−0.03 (−0.22, 0.15)	0.02 (−0.15, 0.18)	0.03 (−0.15, 0.22)
1y	−0.11 (−0.23, 0.01)	−0.05 (−0.18, 0.08)	−0.13 (−0.28, 0.02)
2y	−0.02 (−0.14, 0.10)	0.08 (−0.04, 0.20)	−0.05 (−0.21, 0.10)

*Values are adjusted mean difference (95% confidence interval). Results are from intention-to-treat analyses and reported for patients alive at each time point. At 6m, 1y, and 2y, denominators for Aripiprazole were: 3,530, 3,480, 3,391; Olanzapine: 8,383, 8,244, 8,003; Quetiapine: 8,137, 8,013, 7,809; and Risperidone: 5,941, 5,816, 5,548, respectively. Missing values were replaced using multiple imputation. Models were adjusted for pre-specified baseline covariates: age, sex, ethnicity, SMI diagnosis category, prior use of non-study antipsychotics, level of deprivation (quintile), geographic region, calendar year of index date, number of primary care consultations in prior 6 months, smoking status, comorbidities (alcohol misuse, cerebrovascular disease, diabetes, dyslipidaemia, hypertension, liver disease, myocardial infarction, renal disease, substance misuse), concomitant medications (antidepressants, mood stabilisers, lipid-regulating medications, antidiabetics, antihypertensives), body mass index category, and cardiometabolic values (total cholesterol, LDL-C, HDL-C, triglycerides, systolic blood pressure, diastolic blood pressure, glucose, HbA1c, weight).

mmol/L, millimoles per litre; LDL-C, low-density lipoprotein cholesterol; HDL-C, high-density lipoprotein cholesterol; TC:HDL, total cholesterol to high-density lipoprotein; HbA1c, glycated haemoglobin; kg, kilogram; mmHg, millimetres of mercury; mmol/mol, millimoles per mole.

#### Secondary cardiometabolic outcomes.

Across all three follow-up time-points, we found evidence that patients prescribed aripiprazole had better cardiometabolic outcomes than those prescribed olanzapine for 9 of the 10 parameters (all except glucose), those prescribed quetiapine on 3 (triglycerides, systolic blood pressure, diastolic blood pressure), and those prescribed risperidone on 2 (HDL-C, body weight) (Table 2). Estimates from per-protocol analyses were broadly consistent with these findings (Table J in S1 Appendix).

### Effectiveness

#### Main secondary outcome.

Among the 26,537 patients, 21,440 (80.8%) had linked HES data, of whom 4,306 had at least one psychiatric hospitalisation by 2 years, equating to an unadjusted incidence ranging from 19.0% to 22.1% across treatment groups (Table M in S1 Appendix).

**Fig 2:**
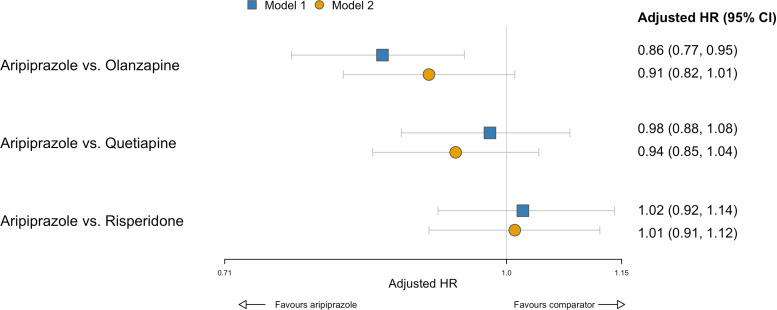
Psychiatric hospitalisation outcome with aripiprazole vs. comparator antipsychotics. HR, hazard ratio. Estimates are cause-specific hazard ratios (95% confidence interval) from intention-to-treat analyses for the effect of aripiprazole versus comparator antipsychotics. Patients were censored at the earliest of first psychiatric hospitalisation, death, completion of 2-years follow-up, end of primary care registration, or administrative censoring. The median (IQR) follow-up time in censored cases was 730 (730–730) days. Analyses are based on 553 events among 2,918 patients in the aripiprazole group, 1,535 events among 6,949 patients in the olanzapine group, 1,252 events among 6,503 patients in the quetiapine group, and 966 events among 5,070 patients in the risperidone group. Model 1 adjusted for pre-specified baseline covariates: age, sex, ethnicity, SMI diagnosis category, prior use of antipsychotics, relative deprivation (quintile), geographic region, calendar year of index date, number of primary care consultations in prior 6 months, smoking status, comorbidities (alcohol misuse, cerebrovascular disease, diabetes, dyslipidaemia, hypertension, liver disease, myocardial infarction, renal disease, substance misuse), concomitant medications (antidepressants, mood stabilisers, lipid-regulating medications, antidiabetics, antihypertensives), body mass index category, and cardiometabolic values (total cholesterol, LDL-C, HDL-C, triglycerides, systolic blood pressure, diastolic blood pressure, glucose, HbA1c, weight). Model 2 adjusted for all of the covariates listed above, with additional adjustment for psychiatric hospitalisation in the prior 2 years. HR, hazard ratio.

**Fig 3:**
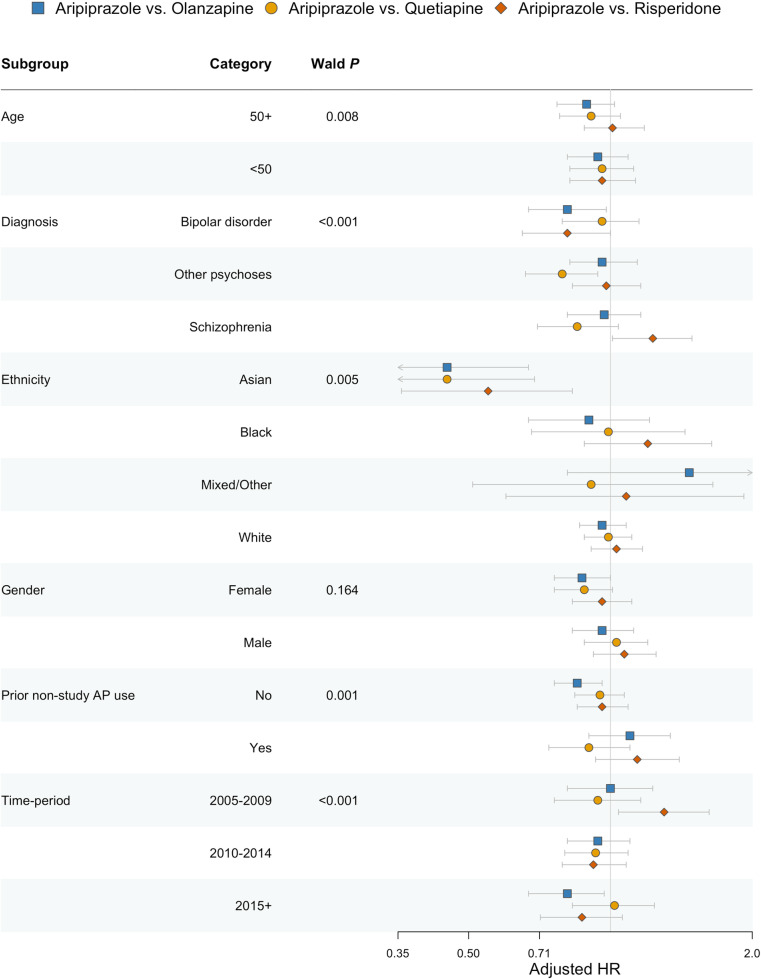
Psychiatric hospitalisation outcome across subgroups. AP, antipsychotic; HR, hazard ratio. The forest plot shows the adjusted hazard ratio of aripiprazole versus each comparator antipsychotic in each subgroup from the intention-to-treat analysis. Estimates to the left of the no effect line (at HR =  1.0) favour aripiprazole while those to the right favour a comparator. Models were adjusted for age, sex, ethnicity, SMI diagnosis category, prior use of antipsychotics, level of deprivation (quintile), geographic region, calendar year of index date, number of primary care consultations in prior 6 months, smoking status, comorbidities (alcohol misuse, cerebrovascular disease, diabetes, dyslipidaemia, hypertension, liver disease, myocardial infarction, renal disease, illicit drug use), concomitant medications (antidepressants, mood stabilisers, lipid-regulating medications, antidiabetics, antihypertensives), body mass index category, cardiometabolic values (total cholesterol, LDL-C, HDL-C, triglycerides, systolic blood pressure, diastolic blood pressure, glucose, HbA1c, weight), and prior psychiatric hospitalisation.

After adjustment for pre-specified confounders, patients prescribed aripiprazole had fewer first psychiatric hospitalisations than those prescribed olanzapine (aHR, 0.86, 95% CI, 0.77 to 0.95, *p* = 0.003); however, this difference attenuated after adjusting for prior hospitalisation (aHR, 0.91, 95% CI, 0.82 to 1.01, *p* = 0.078) (Fig 2). After adjusting for prior hospitalisation, patients prescribed aripiprazole had similar hospitalisation rates as those prescribed quetiapine (aHR, 0.94, 95% CI, 0.85 to 1.04, *p* = 0.230) and risperidone (aHR, 1.01, 95% CI, 0.91 to 1.12, *p* = 0.854), estimates without additional adjustment for prior hospitalisation were comparable (Fig 2). We found evidence of heterogeneity in these results across subgroups based on SMI diagnosis (interaction *p* < 0.001), age (*p* = 0.008), ethnicity (*p* = 0.005), prior antipsychotic use (*p* = 0.001), and time period (*p* < 0.001), but not sex (*p* = 0.164) (Fig 3). Estimates from analyses using a more restrictive outcome definition, accounting for death as a competing risk, using inverse probability of treatment weighting, accounting for general practice clustering, and per-protocol (with and without censoring weighting) were all broadly similar to those from the primary analyses (Tables K and N in S1 Appendix); however, in the inverse probability of treatment weighted per-protocol analysis, patients prescribed aripiprazole had lower hospitalisation rates than comparators.

#### Other secondary effectiveness outcomes.

Among the 26,537 patients, 9,245 antipsychotic discontinuation events and 1,786 deaths were observed in 2 years. At 2 years, the unadjusted incidence of discontinuation ranged from 36.7% to 38.0%, while mortality ranged from 5.20% to 9.51%, across treatment groups (Table M in S1 Appendix). After confounder adjustment, patients prescribed aripiprazole had similar rates of discontinuation and mortality to the comparators (Table O in S1 Appendix).

## Discussion

In this target trial emulation involving 26,537 patients, we found little evidence of differences in total cholesterol levels after 1 year or in the rate of psychiatric hospitalisation among patients prescribed aripiprazole when compared to those prescribed olanzapine, quetiapine, or risperidone. However, patients prescribed aripiprazole had better outcomes on other cardiometabolic parameters, such as body weight and blood pressure. These results suggest that aripiprazole may confer some cardiometabolic benefits over the comparators without compromising psychiatric effectiveness.

Contrary to our hypothesis, patients prescribed aripiprazole had similar total cholesterol levels to the comparators 1 year after the first prescription. CIs were narrow, suggesting clinically important differences are unlikely. These findings were robust—similar results were seen in sensitivity analyses. There were little differences in the proportions prescribed lipid-regulating medications across treatment groups during follow up. We did; however, find evidence that patients prescribed aripiprazole had lower total cholesterol after 6 months when compared to those prescribed olanzapine. Network meta-analyses reported that patients randomised to aripiprazole had lower total cholesterol than olanzapine and quetiapine during acute (median duration, 6 weeks) [4] and mid- to long-term (45 weeks) [3] treatment of schizophrenia. Although the latter should, in theory, be comparable to the duration of our primary outcome—four of the five studies providing direct evidence comparing aripiprazole versus olanzapine lasted ≤ 28 weeks (no direct evidence was included for our other comparisons).

Across all 10 cardiometabolic safety outcomes and 3 follow-up time points, where evidence of differences was found, all estimates favoured aripiprazole. In particular, patients prescribed olanzapine, the most frequently prescribed antipsychotic in the UK [7], had the least favourable outcomes of the comparators. Although some estimates might be considered modest in effect size, the cumulative burden of multiple cardiometabolic abnormalities is likely to confer significant cardiovascular risk, especially at the population level. Future studies are needed to investigate the comparative risks of major adverse cardiovascular events.

Psychiatric hospitalisation, encompassing efficacy, tolerability, and adherence [36], was our main effectiveness outcome. We hypothesised aripiprazole would have effectiveness similar to the comparators, but found evidence of lower hospitalisation rates with aripiprazole compared to olanzapine. However, this may be due to the baseline difference in proportions previously hospitalised (olanzapine, 35%; other antipsychotics, 24%–28%)—the hazard ratio attenuated after accounting for the imbalance, but the CI remained largely in favour of aripiprazole (0.82–1.01). Nevertheless, our results do not indicate an increased risk of hospitalisation with aripiprazole—and are consistent with a recent network meta-analysis of RCTs investigating maintenance treatments in schizophrenia (but CIs in the meta-analysis were very wide for all comparators, with few direct comparisons) [2].

This study has several strengths. First, we reduced bias by adopting the target trial emulation framework—emulating a hypothetical trial unlikely ever to be undertaken at the same scale and applying minimal exclusions to help ensure relevance to the target population. Second, we included a large, powered, diverse sample, followed over 2 years, from 2,163 primary care practices across the UK, with data reflecting real-world practice. We studied safety and effectiveness in the same cohort, enabling a direct comparison of risks and benefits. Third, we conducted intention-to-treat and per-protocol analyses—essential given a high incidence of discontinuation was expected and observed (37%–38% by 2 years). We also consulted with lived experience advisors and focussed the study on unwanted effects which impact quality of life. Finally, we studied the real-world clinical dilemma of antipsychotic choice, but future studies are needed to evaluate other aspects of antipsychotic treatment, such as dose and adherence.

This study also has limitations. First, although all patients were likely prescribed antipsychotics to manage SMI symptoms, we observed baseline differences across treatment groups, highlighting differential prescribing. Following causal inference methods, we adjusted for many observed potential confounders. We also undertook inverse probability of treatment weighting sensitivity analyses, which may be methodologically preferable with many confounders. However, residual confounding may remain due to unmeasured confounders (e.g., illness severity, health behaviours, genetics, patient, and clinician preferences). In particular, psychiatric illness severity is challenging to measure. Although we adjusted for several covariates associated with severity (e.g., diagnosis, prior antipsychotic use, concomitant psychiatric medications, and prior hospitalisation), we could not consider functional impairment and psychiatric service contacts as these were not recorded in our data source.

The key advantage of an RCT is that, on average and given sufficient sample size, randomisation generally ensures that comparison groups are balanced on both observed and unobserved covariates. Although target trial emulation improves transparency and reduces some methodological biases—and therefore offers advantages over other observational designs—the statistical models used require the unverifiable assumption of no unmeasured confounding in the treatment–outcome relationship, necessitating rich data sources with sufficient measures of potential confounders. One way to assess the success of this emulation might have been to compare our results with those obtained from RCTs directly [37]. However, this is not directly possible because no RCTs closely match our study’s population, setting, and longer-term outcomes and the known limitations of antipsychotic RCTs [13]. We, therefore, tentatively compared our results with those from syntheses of RCT evidence. Reassuringly, we replicated (up to 1 year) the well-known increased body weight observed with olanzapine versus aripiprazole [2–4,8–11].

Second, our sample was broadly representative of the target population, but underrepresented younger patients and those diagnosed with schizophrenia, possibly reflecting healthcare utilisation patterns. Third, although most long-term antipsychotic prescribing in the UK occurs in primary care, we may have missed a smaller number of prescriptions issued in secondary care (e.g., for inpatients), resulting in misclassification. Fourth, there were differences in starting doses at baseline, so we emulated a pragmatic trial with dosage at clinical discretion, as in real-world practice. Finally, despite guidelines for health checks in primary care, there was a high level of missing follow-up cardiometabolic outcomes, but ≥ 80% contributed data for at least one time point for most outcomes. Although we used multiple imputation to handle missing data, these results may not generalise to people who engage less, or not at all, with primary care. The missing data importantly shows that many patients were not regularly monitored, despite established guidelines and financial incentives for primary care practices.

## Conclusions

Data from our large, powered, diverse, real-world target trial emulation sample, followed over 2 years, suggest that adults diagnosed with severe mental illness prescribed aripiprazole have similar total cholesterol 1 year after first prescription compared to those prescribed olanzapine, quetiapine, and risperidone. However, patients prescribed aripiprazole had better outcomes on some other cardiometabolic parameters, and there was little evidence of differences in effectiveness. Our findings inform a common clinical dilemma and contribute to the evidence base for real-world clinical decision-making on antipsychotic choice for patients diagnosed with severe mental illness.

## Supporting information

S1 Protocol Pre-registered study protocol. (PDF)

S1 STROBE Checklist STROBE checklist. (DOCX)

S1 Appendix Supplementary tables and figures. (DOCX)

## Abbreviations

aHR: adjusted hazard ratio
aMD: adjusted mean difference
ANOVA: analysis of variance
CIs: confidence intervals
CPRD: Clinical Practice Research Datalink
HbA1c: glycated haemoglobin
HDL-C: high-density lipoprotein cholesterol
HES: Hospital Episode Statistics
LDL-C: low-density lipoprotein cholesterol
ONS: Office for National Statistics
RCT: randomised clinical trial
SMI: severe mental illness
STROBE: Strengthening the Reporting of Observational Studies in Epidemiology
