# Supplementary material for: Comparative cardiometabolic safety and effectiveness of aripiprazole in people with severe mental illness: A target trial emulation
Source: PLoS Med. 2025 Jan 23;22(1):e1004520. doi: 10.1371/journal.pmed.1004520 (PMC11778676; doi:10.1371/journal.pmed.1004520)
Supplement: S1 Protocol — (PDF) [file pmed.1004520.s001.pdf]

Balancing cardiometabolic risks and psychiatric effectiveness of commonly prescribed antipsychotics in UK primary care: Protocol for a head-to-head target trial emulation study

---

Alvin Richards-Belle, Division of Psychiatry, University College London

CONTENTS

***Background and rationale* ... 2**

Aims ... 2

Hypotheses ... 2

***Methods* ... 3**

Study design ... 3

Setting ... 4

Data source ... 4

Population ... 4

Inclusion criteria ... 4

Exclusion criteria ... 4

Recruitment and follow-up period ... 4

Exposures ... 5

Outcomes ... 5

Cardiometabolic outcomes ... 5

Effectiveness outcomes ... 6

***Statistical methods* ... 7**

Sample size ... 7

Missing data ... 7

Descriptive statistics ... 7

Primary analyses ... 8

Principles ... 8

Cardiometabolic outcomes ... 9

Effectiveness outcomes ... 9

Subgroup analysis ... 9

Sensitivity and other analyses ... 9

***References* ... 10**

Background and rationale

Antipsychotic medications are a mainstay of treatment in severe mental illness (SMI) (e.g., schizophrenia, bipolar disorder and other psychotic illnesses). They are effective at treating symptoms, particularly positive psychotic symptoms, for many people - and can be life-saving for some.[1–4] However, as with all medications, their use is not without risks. Adverse effects can often include weight gain, raised cholesterol levels and insulin resistance,[5] which increase risks of cardiovascular and metabolic diseases and subsequent premature mortality.[6–8] The decision, therefore, to prescribe one medication over another requires a careful evaluation of potential benefits and risks for each individual, informed by robust, high-quality evidence.

One promising approach to evaluate longer term antipsychotic outcomes is target trial emulation (TTE).[9] Leveraging a large, national database of routine, electronic, health records, this study will aim to demonstrate the utility of the TTE approach in comparing risks and benefits of commonly prescribed antipsychotics in UK primary care. This document details the pre-specified study protocol, including analysis plan.

The clinical research question is: in people with a diagnosis of SMI prescribed antipsychotic medications in primary care [population], does aripiprazole [intervention], as compared to three other commonly prescribed antipsychotics (olanzapine, risperidone, quetiapine) [comparators], have a better cardiometabolic profile without an increased risk of ineffectiveness (i.e., psychiatric hospitalisation, mortality, discontinuation) [outcomes]?

Aims

- To estimate the causal effect of commonly prescribed antipsychotic medications on short- and longer-term cardiometabolic and effectiveness outcomes in patients with SMI.

- To demonstrate the utility of the TTE approach in comparing risks and benefits of commonly prescribed antipsychotics in UK primary care.

Hypotheses

The hypotheses are that: (1) aripiprazole will have the most favourable cardiometabolic profile (and olanzapine the least); and (2) all four antipsychotics will be equivalent in terms of their effectiveness.

Methods

Study design

An observational, electronic health record-embedded, head-to-head, pragmatic, target trial emulation study. A comparison of a hypothetical ideal and emulation target trial design features is shown in Table 1.

Table 1. Comparison of a hypothetical ideal and emulation target trial design features.*

Trial design	Hypothetical ideal target trial	Target trial emulation
Eligibility criteria	• Aged 18 to 99 years.<br>• Severe mental illness diagnosis.<br>• Prescribed either oral aripiprazole, olanzapine, risperidone, or quetiapine in primary care.<br>• Informed consent to participate and have a baseline blood test.	• Aged 18 to 99 years.<br>• Severe mental illness diagnosis recorded in primary care.<br>• Prescribed either oral aripiprazole, olanzapine, risperidone, or quetiapine in primary care.<br>• Baseline blood test result for lipids and/or HbA1c recorded in primary care.
Recruitment period	2005-2017	2005-2017
Follow-up duration	24 months	24 months
Outcome(s)	• Primary cardiometabolic: Total cholesterol<br>• Main effectiveness: Psychiatric hospitalisation	• Primary cardiometabolic: Total cholesterol<br>• Main effectiveness: Psychiatric hospitalisation[a]
Treatments strategies	• Aripiprazole<br>• Olanzapine<br>• Quetiapine<br>• Risperidone	• Aripiprazole<br>• Olanzapine<br>• Quetiapine<br>• Risperidone
Assignment procedures	Randomisation	Non-randomisation
Causal contrasts of interest	Intention-to-treat (primary) and per-protocol (sensitivity)	Intention-to-treat (primary) and per-protocol (sensitivity)
Estimands	Average treatment effect	Average treatment effect
Analysis plan	Adjusted linear and cox regression models	Adjusted linear and cox regression models

* Table adapted from Hernan and Robins (2016)[9]

[a] Only available in a subset of participants

Setting

UK primary care.

Data source

Clinical Practice Research Datalink (CPRD) Gold and Aurum databases, linked to Hospital Episode Statistics (HES) and Office for National Statistics (ONS) Death Registrations.

Population

Patients with a diagnosis of SMI prescribed antipsychotic medications in primary care between 2005 and 2017. The patient index date is the date on which the patient is first prescribed an antipsychotic of interest in the study period.

Inclusion criteria

- Aged 18 to 99 years.
- Severe mental illness (i.e., schizophrenia, bipolar disorder or other non-affective psychotic illness) diagnosis recorded in primary care.
- First-time prescription of either oral aripiprazole, olanzapine, risperidone, or quetiapine in primary care.
- Results of at least one blood test for lipids or or glycated hemoglobin recorded in primary care in the prior two years.

Exclusion criteria

- Less than six months of primary care registration on index date.
- Deregistered with primary care practice on index date.
- Prescribed aripiprazole, olanzapine, risperidone, or quetiapine in primary care prior to study period.
- Prescribed aripiprazole, olanzapine, risperidone, or quetiapine as a long-acting injectable on index date.
- Prescribed more than one antipsychotic medication in primary care on index date (or in the last 90 days, for long acting injectables).
- Dementia diagnosis recorded in primary care on or prior to index date.

Recruitment and follow-up period

Patients fulfilling the eligibility criteria will enter the study on the date (index date) on which they are first prescribed the antipsychotic of interest between the year 2005 (the first full calendar year in which aripiprazole was available in the UK) up to and including March 2017. Study patients will exit the study at the earliest of: completion of follow-up (24 months); end of primary care

registration; death; or administrative censoring. Limiting entry at March 2017 allows accrual of two years follow-up time for the final included patients based on the CPRD data currently available for this study. A STROBE flow chart[10] will display how the cohort was derived.

Variables will be considered baseline if measured on the index date or within the two years prior, with the most recent used, where multiple are available. Baseline diagnoses will be characterised using the patients entire prior primary care medical history available at baseline (with an allowance for recording delays in the case of SMI diagnoses).

Exposures

The exposure is defined as the first receipt of an antipsychotic of interest during the recruitment period. Aripiprazole, olanzapine, risperidone, and quetiapine were selected as the focus of this study as they were previously identified as the top four most frequently prescribed antipsychotics to patients with SMI in primary care between 2010-2019.

With regards to SMI diagnosis and prior receipt of antipsychotics, this cohort will approximate those typically included in most clinical trials of antipsychotics, whereby diagnosis typically occurred sometime in the past, and some patients have already been exposed to antipsychotics.[11]

Outcomes

The primary outcome is the total cholesterol level at 12 months. Of the secondary outcomes, the most important is psychiatric hospitalisation, also at 12 months.

Cardiometabolic outcomes

- Total cholesterol (mmol/L) [primary]
- Low-density lipoprotein cholesterol (LDL-C) (mmol/L)
- High-density lipoprotein cholesterol (HDL-C) (mmol/L)
- Triglycerides (mmol/L)
- Total cholesterol to HDL-C ratio
- Systolic blood pressure (mm Hg)
- Diastolic blood pressure (mm Hg)
- Glucose (mmol/L)
- Glycated hemoglobin (HbA1c) (mmol/mol)
- Body weight (kg)

Cardiometabolic outcomes will be reported at 6, 12 and 24 months. As data are collected for clinical purposes, actual measurement time will vary across participants. Time-windows for cardiometabolic outcomes will be widened to include a period before and after the study outcome time-point (e.g., at 6 ± 3 months, 12 ± 3 months and 24 ± 6 months), with the measurement closest to the study time-point used. Clinical parameters were chosen as the main outcomes, instead of diagnoses, in order to estimate the magnitude of the impact of antipsychotics.

Effectiveness outcomes

Effectiveness outcomes will be analysed using time-to-event methods and reported at 6, 12 and 24 months.

- Psychiatric hospitalisation

This outcome was chosen as an indicator of treatment failure. It is noted that this outcome will only pick up potentially severe cases of treatment failure (treatment failure resulting in less severe outcomes is another important outcome but unavailable in CPRD). This outcome was included to inform the risk-benefit ratio of antipsychotics (i.e., if an antipsychotic is associated with a better cardiometabolic profile, but a significantly greater risk of hospitalisation, it may not be considered acceptable).

- All-cause discontinuation

All-cause discontinuation of the prescribed/allocated antipsychotic is considered an important outcome encompassing both patients' and clinicians' judgements of effiectivness, safety and tolerability in a global measure,[12] frequently used in clinical trials of antipsychotics.[11] This outcome was included to enable comparison with such trials.

- All-cause mortality

This outcome was included to inform the risk-benefit ratio of antipsychotics (i.e., if an antipsychotic is associated with a better cardiometabolic profile, but a significantly greater risk of mortality, it would not be considered acceptable).

Statistical methods

Sample size

A formal power calculation will not be conducted as all patients meeting the eligibility criteria will be included, however an illustrative power calculation for the primary outcome (total cholesterol) is shown in Table 2.

Table 2. Illustrative power calculation.*

	N per group	
**Delta**	*For 80% power*	*For 90% power*
0.25	393	526
0.20	614	822
0.15	1091	1460
0.10	2454	3285

* *Assuming a standard deviation of 1.25[13] and a significance level of 0.05.*

Missing data

Although physical health checks for patients with SMI have been incentivised in primary care since 2004, operationalisation and specific criteria have varied significantly over time - therefore, a relatively high level of missing data is anticipated for cardiometabolic outcomes, particularly those requiring a blood test for measurement.[14,15] We aim to mitigate this issue at baseline to some extent through use of an inclusion criterion requiring either lipid or HbA1c test results recorded at baseline. Missing individual cardiometabolic parameters will be calculated based on other observed values from a given test date (e.g. calculation of LDL-C using the Friedewald formula) to ensure data are as complete as possible. We will then perform multiple imputation to impute missing values, conditioned on the observed values. Baseline characteristics of patients with and without missing outcomes will be compared to examine if there are any observable systematic reasons for missingness to inform imputation strategies. A complete-case analysis will also be conducted as a sensitivity analyses.

Descriptive statistics

Descriptive statistics will summarise baseline characteristics, stratified by treatment group, including: age at index date, sex, ethnicity, SMI diagnosis, age at diagnosis, geographic region, level of deprivation (quintile), body mass index (BMI), physical health comorbidities (e.g., dyslipipaedmia, diabetes, hypertension, cerebrovascular disease, myocardial infaction, liver disease, renal disease), smoking status, alcohol misuse, substance misuse, and concomitant medications (e.g., lipid regulating medications, antihypertensives, antidiabetics, antidepressants).

Descriptives of each of the baseline cardiometabolic parameters and the number of psychiatric hospitalisations within the two years prior will also be presented. Exposure to antipsychotics, including the number of prescriptions, dose (in raw and equivalent units) and follow-up time, will also be summarised.

Summary measures will be mean and standard deviation for continuous (approximately) normally distributed variables, medians and interquartile ranges for non-normally distributed variables, and frequencies and percentages for categorical variables. The level of missing data for each variable will be indicated.

Primary analyses

Principles

An intention-to-treat (observational analog) approach will be taken in the primary analyses, with patients analysed in their original treatment group, irrespective of whether they continued to be prescribed the original antipsychotic. Patients will only be included in the cohort once. Aripiprazole will be compared with each comparator antipsychotic, such that all treatment effects are interpreted with reference to aripiprazole. All analyses will be conducted in R (version 4.1.2).

In addition to unadjusted analyses, outcome models will be adjusted for baseline covariates. Covariates were identified through literature review and clinical experience, and include: age, sex, ethnicity, level of deprivation (quintile), geographic region, calendar year of index date, number of primary care contacts in the prior six months, smoking status, alcohol misuse, substance misuse, physical health diagnoses (dyslipidaemia and selected diagnoses from the Charlson Comorbidity Index and the Elixhauser Comorbidity Index [e.g., diabetes, hypertension, cerebrovascular disease, myocardial infaction, renal failure, liver disease]), prior use of antipsychotics, relevant concomitant baseline medications (i.e., lipid regulating medications, antihypertensives, antidiabetics, antidepressants, mood stabiliers) and baseline cardiometabolic values (e.g., total cholesterol, LDL-C, HDL-C, trigylcerides, systolic blood pressure, diastolic blood pressure, glucose, HbA1c, weight, BMI category). Results from adjusted analyses using the multiply imputed datasets will be considered the primary effect estimates.

Confidence intervals and p-values

All applicable statistical tests will be two-sided, use a 5% significance level and reported with 95% confidence intervals (CIs). Clinical significance of any effects will be determined through interpreting effect sizes and CIs. *P* values will not be adjusted for multiple comparisons because we specified a primary outcome and will interpret the results for the secondary outcomes judiciously in light of multiple testing (noting that results for cardiometabolic outcomes are expected to be associated).

Cardiometabolic outcomes

Linear regression will be used to model the association of each antipsychotic with each cardiometabolic parameter as a continuous outcome at each time point, unadjusted and adjusted for the variables mentioned previously.

Effectiveness outcomes

Time-to-event analysis, including Kaplan-Meier methods and cox proportional hazards regression, will be used to model the time to discontinuation, time to psychiatric hospitalisation, and time to death, unadjusted and adjusted for the variables mentioned previously. Models for discontinuation and psychiatric hospitalisation will account for the competing risk of death. Probabilities of all outcomes will be reported at 6, 12 and 24 months, appropriately handling censoring for patients that are lost to follow-up. Absence of a record for a hospitalisation will be assumed to represent that there was no admission. Absence of a recorded death date will be assumed to represent that the patient survived.

Psychiatric hospitalisation data will only be available for a subset of the overall study population (i.e., participants registered at participating CPRD GP practices in England and who were eligible for linkage to HES).

Subgroup analysis

Heterogeneity in results will be explored via a limited number of subgroup analyses. Subgroup defining variables will include: age categories, sex, ethnicity, SMI diagnosis, prior use of antipsychotics, and time periods. Interaction tests will be used to determine the significance of any subgroup effects.

Sensitivity and other analyses

In a “per-protocol” analysis, the primary analyses will be repeated, but patients will be censored if they switch to one of the other antipsychotics of interest (so that outcomes can be more easily attributed to a particular medication) or at three months following the last prescription date of the antipsychotic of interest (to allow for later development of adverse effects or delayed reported).[16]

In a “weighted” analysis, we will use weighted propensity score methods as an alternative approach to handling imbalances in measured baseline confounders. This will use inverse probability of treatment weighting (IPTW) to estimate average treatment effects. IPTW creates a pseudo-population weighted by the inverse of the propensity score, aiming to achieve a sample within which treatment groups are more comparable with respect to measured baseline characteristics and thereby reducing confounding bias. Propensity scores will be estimated by including all adjustment variables mentioned previously. Balance, in terms of standardised mean

differences, will be compared before and after IPTW to observe the success of covariate balance. Interactions and non-linear forms will be considered to improve specification of the propensity score iteratively,[17] as necessary. Potential extreme weights will be dealt with through stabilisation or truncation. 'Doubly robust' methods will be considered if imbalances remain (e.g., inclusion of the imbalanced variable as a covariate in outcome models).[18]

We will also explore the impact of alternative eligibility criteria (i.e., not directly following the TTE approach) in order to identify and evaluate potential sources of bias.

References

1. Tiihonen, J., Mittendorfer-Rutz, E., Torniainen, M., Alexanderson, K. & Tanskanen, A. Mortality and Cumulative Exposure to Antipsychotics, Antidepressants, and Benzodiazepines in Patients With Schizophrenia: An Observational Follow-Up Study. *AJP* **173**, 600–606 (2016).
2. Tiihonen, J. *et al.* Effectiveness of antipsychotic treatments in a nationwide cohort of patients in community care after first hospitalisation due to schizophrenia and schizoaffective disorder: observational follow-up study. *BMJ* **333**, 224 (2006).
3. Tiihonen, J. *et al.* 11-year follow-up of mortality in patients with schizophrenia: a population-based cohort study (FIN11 study). *The Lancet* **374**, 620–627 (2009).
4. Ran, M.-S. *et al.* Different outcomes of never-treated and treated patients with schizophrenia: 14-year follow-up study in rural China. *Br J Psychiatry* **207**, 495–500 (2015).
5. Pillinger, T. *et al.* Comparative effects of 18 antipsychotics on metabolic function in patients with schizophrenia, predictors of metabolic dysregulation, and association with psychopathology: a systematic review and network meta-analysis. *Lancet Psychiatry* **7**, 64–77 (2020).
6. Firth, J. *et al.* The Lancet Psychiatry Commission: a blueprint for protecting physical health in people with mental illness. *The Lancet Psychiatry* **6**, 675–712 (2019).
7. Hayes, J. F., Marston, L., Walters, K., King, M. B. & Osborn, D. P. J. Mortality gap for people with bipolar disorder and schizophrenia: UK-based cohort study 2000–2014. *The British Journal of Psychiatry* **211**, 175–181 (2017).

8. Osborn, D. P. J. *et al.* Relative Risk of Cardiovascular and Cancer Mortality in People With Severe Mental Illness From the United Kingdom's General Practice Research Database. *Archives of General Psychiatry* **64**, 242–249 (2007).
9. Hernán, M. A. & Robins, J. M. Using Big Data to Emulate a Target Trial When a Randomized Trial Is Not Available. *Am J Epidemiol* **183**, 758–764 (2016).
10. Vandenbroucke, J. P. *et al.* Strengthening the Reporting of Observational Studies in Epidemiology (STROBE): Explanation and Elaboration. *PLOS Medicine* **4**, e297 (2007).
11. Lieberman, J. A. *et al.* Effectiveness of Antipsychotic Drugs in Patients with Chronic Schizophrenia. *New England Journal of Medicine* **353**, 1209–1223 (2005).
12. Mustafa, S. *et al.* Predictors of 'all-cause discontinuation' of initial oral antipsychotic medication in first episode psychosis. *Schizophrenia Research* **201**, 287–293 (2018).
13. Richards-Belle, A. *et al.* Associations of antidepressants and antipsychotics with lipid parameters: Do CYP2C19/CYP2D6 genes play a role? A UK population-based study. *J Psychopharmacol* 2698811231152748 (2023) doi:10.1177/02698811231152748.
14. Osborn, D. P. J. *et al.* Cardiovascular risk prediction models for people with severe mental illness: results from the prediction and management of cardiovascular risk in people with severe mental illnesses (PRIMROSE) research program. *JAMA Psychiatry* **72**, 143–151 (2015).
15. Bosanquet, K. Meeting the physical health needs of people with serious mental illness in primary care. (University of York, 2020).
16. Hayes, J. F. *et al.* Adverse Renal, Endocrine, Hepatic, and Metabolic Events during Maintenance Mood Stabilizer Treatment for Bipolar Disorder: A Population-Based Cohort Study. *PLOS Medicine* **13**, e1002058 (2016).
17. Ali, M. S. *et al.* Propensity Score Methods in Health Technology Assessment: Principles, Extended Applications, and Recent Advances. *Frontiers in Pharmacology* **10**, (2019).
18. McCaffrey, D. F. *et al.* A tutorial on propensity score estimation for multiple treatments using generalized boosted models. *Statistics in Medicine* **32**, 3388–3414 (2013).
