# Supplementary material for: Comparative cardiometabolic safety and effectiveness of aripiprazole in people with severe mental illness: A target trial emulation
Source: PLoS Med. 2025 Jan 23;22(1):e1004520. doi: 10.1371/journal.pmed.1004520 (PMC11778676; doi:10.1371/journal.pmed.1004520)
Supplement: S1 Appendix — (DOCX) [file pmed.1004520.s003.docx]

**Supporting Information**

**“Comparative cardiometabolic safety and effectiveness of aripiprazole
in people with severe mental illness: A target trial emulation”**

Alvin Richards-Belle, Naomi Launders, Sarah Hardoon, Al Richards, Kenneth K.C. Man, Neil M. Davies, Elvira Bramon, Joseph F. Hayes, David P.J. Osborn

**Content**

[Table A. Psychiatric hospitalisation definition and ICD-10 codes. 2](#_Toc186714246)

[Table B. Reference ranges for cardiometabolic values. 3](#_Toc186714247)

[Table C. Covariates. 4](#_Toc186714248)

[Table D. Variables included in the imputation model. 6](#_Toc186714249)

[Table E. Illustrative power calculation for primary outcome.* 7](#_Toc186714250)

[Table F. Characteristics of study cohort compared to patients prescribed a study antipsychotic during the study period but who did not meet eligibility criteria. 8](#_Toc186714251)

[Table G. Unadjusted cardiometabolic parameters at baseline and at 6m, 1y, and 2y follow-up.* 9](#_Toc186714252)

[Table H. Proportion of patients with observed cardiometabolic parameter data.* 11](#_Toc186714253)

[Table I. Comparison of baseline characteristics of patients with and without observed primary outcome data.* 13](#_Toc186714254)

[Table J. Cardiometabolic outcomes with aripiprazole versus comparator antipsychotics – intention-to-treat and per-protocol results.* 15](#_Toc186714255)

[Table K. Additional sensitivity analyses for the main outcomes. 17](#_Toc186714256)

[Table L. Lipid-regulating medication prescriptions after index date.* 19](#_Toc186714257)

[Table M. Unadjusted estimated probabilities of each effectiveness outcome. 20](#_Toc186714258)

[Table N. Psychiatric hospitalisation outcome with aripiprazole versus comparator antipsychotics – intention-to-treat and per-protocol results. 21](#_Toc186714259)

[Table O. Discontinuation and mortality outcomes with aripiprazole versus comparator antipsychotics – intention-to-treat and per-protocol results. 22](#_Toc186714260)

[Figure A. Directed acyclic graph for the baseline confounding structure for cardiometabolic safety outcomes. 23](#_Toc186714261)

[Figure B. Inverse probability of treatment weighting sensitivity analysis - covariate balance plot. 24](#_Toc186714262)

[Figure C. Inverse probability of treatment weighting sensitivity analysis - distribution of weights. 25](#_Toc186714263)

[Figure D. Total cholesterol at one year outcome across subgroups. 26](#_Toc186714264)

[Supporting References 27](#_Toc186714265)

Table A. Psychiatric hospitalisation definition and ICD-10 codes.

| **Outcome** | **Type** | **Primary diagnosis?** | **ICD-10 codes** |
| --- | --- | --- | --- |
| Psychiatric hospitalisation | Admission associated with a psychiatric diagnosis code as primary diagnosis | Primary | Any ICD-10 code containing “F” e.g., F20, F30 |
|  | Admission associated with an intentional self-harm diagnosis code | Primary or not primary* | X60, X61, X62, X63, X64, X65, X66, X67, X68, X69, X70, X71, X72, X73, X74, X75, X76, X77, X78, X79, X80, X81, X82, X83, X84, Y87.0 |

ICD-10, International Classification of Diseases 10th Revision.

* We considered both primary and not primary diagnoses for intentional self-harm related admissions in order to improve sensitivity for self-harm related admissions as other codes relating to the self-harm event are typically recorded as primary diagnoses e.g., poisoning or injury.

Table B. Reference ranges for cardiometabolic values.

| **Parameter** | **Lower** | **Upper** |
| --- | --- | --- |
| Systolic blood pressure (mm Hg) | 52.2 | 269.5 |
| Diastolic blood pressure (mm Hg) | 27.9 | 167.2 |
| Weight (kg) | 27.5 | 250 |
| Total cholesterol (mmol/L) | 1.75 | 21.0 |
| LDL-C (mmol/L) | 0.26 | 10.30 |
| HDL-C (mmol/L) | 0.3 | 4.7 |
| Triglycerides (mmol/L) | 0.10 | 40.0 |
| TC:HDL ratio | 1 | 70 |
| Glucose (mmol/L) | 0.6 | 45 |
| HbA1c (mmol/mol) | 19.13 | 304.17 |

LDL-C, low-density lipoprotein cholesterol; HDL-C, high-density lipoprotein cholesterol; TC:HDL, total cholesterol to high-density lipoprotein; HbA1c, glycated haemoglobin; kg, kilogram; mm Hg, millimetres of mercury; mmol/mol, millimoles per mole.

Recorded values outside of these reference ranges were deemed implausible and set to missing.

Table C. Covariates.

| **Variable** | **Type** | **Categories** |
| --- | --- | --- |
| **Demographics** |  |  |
| Age at index date | Continuous | - |
| Sex | Binary | Male, Female |
| Ethnicity^1^ | Categorical | Asian, Black, Mixed, Other, White |
| Geographic region (of primary care practice) | Categorical | East Midlands, East of England, London, North East, North West, Northern Ireland,  Scotland, South East, South West, Wales, West Midlands, Yorkshire & The Humber |
| Relative deprivation | Categorical | Quintile of the 2019 English Index of Multiple Deprivation |
| **Psychiatric history** |  |  |
| SMI diagnosis | Categorical | Bipolar disorder, Other non-organic psychoses, Schizophrenia |
| Prior use of non-study antipsychotics | Binary | Yes, No |
| **Comorbidities^2^** |  |  |
| Alcohol misuse | Binary | Yes, No |
| Cerebrovascular disease | Binary | Yes, No |
| Diabetes | Binary | Yes, No |
| Dyslipidaemia | Binary | Yes, No |
| Hypertension | Binary | Yes, No |
| Liver disease | Binary | Yes, No |
| Myocardial infarction | Binary | Yes, No |
| Renal disease | Binary | Yes, No |
| Substance misuse | Binary | Yes, No |
| **Prescribed concomitant medications^3^** |  |  |
| Antidepressants | Binary | Yes, No |
| Antidiabetics | Binary | Yes, No |
| Antihypertensives | Binary | Yes, No |
| Lipid-regulating medications | Binary | Yes, No |
| Mood stabilisers | Binary | Yes, No |
| **Cardiometabolic values^4^** |  |  |
| Total cholesterol | Continuous | - |
| LDL-C | Continuous | - |
| HDL-C | Continuous | - |
| Triglycerides | Continuous | - |
| Systolic blood pressure | Continuous | - |
| Diastolic blood pressure | Continuous | - |
| Glucose | Continuous | - |
| HbA1c | Continuous | - |
| Body weight | Continuous | - |
| Body mass index category | Categorical | Underweight, Healthy, Overweight, Obese |
| **Other** |  |  |
| Calendar year of index date | Continuous | - |
| Number of primary care consultations in prior 6m | Continuous | - |
| Smoking status | Categorical | Never smoked, Ex-smoker, Current smoker |
| **Post-hoc** |  |  |
| Psychiatric hospitalisation (in the 2y prior to index date) | Binary | Yes, No |

SMI, severe mental illness; LDL-C, low-density lipoprotein cholesterol; HDL-C, high-density lipoprotein cholesterol; HbA1c, glycated haemoglobin.

^1^ For patients without ethnicity coded in CPRD, ethnicity data were sourced from linked HES data, where available. Where a patient had multiple ethnicity categories recorded, the most frequently recorded was used, or the most recent, if frequencies were equal.

^2^ Defined according to diagnostic codes recorded on or prior to the index date.

^3^ Defined according to product codes recorded on or within the two years prior to the index date.

^4^ Defined according to test result values recorded on or within the two years prior to the index date, using the value closest to the index date. For values requiring a blood test for measurement, values recorded up to seven days after the index date were also considered on the assumption that results might relate to the date on which test results were received, rather than the date on which the blood test was taken.

Table D. Variables included in the imputation model.

| **Category** | **Variables** |
| --- | --- |
| Baseline covariates | - Treatment group - Age at index date - Sex - Ethnicity - SMI diagnosis - Prior use of non-study antipsychotics - Patient-level Index of Multiple Deprivation (quintile) - Practice-level Index of Multiple Deprivation (quintile) - Geographic region (of primary care practice) - Index year - Number of primary care consultations in prior 6m - Smoking status - Comorbidity indicators: alcohol misuse, substance misuse, dyslipidaemia, diabetes, hypertension, cerebrovascular disease, myocardial infarction, renal disease, and liver disease. - Concomitant medication indicators: lipid-regulation medications, antihypertensives, antidiabetics, antidepressants, and mood stabilisers. - Baseline cardiometabolic parameters: Total cholesterol, LDL-C, HDL-C, triglycerides, TC:HDL ratio, body weight, systolic blood pressure, diastolic blood pressure, glucose, HbA1c |
| Auxiliary variables | - Most recent SMI diagnosis - Age at first SMI diagnosis - Age at first antipsychotic prescription - Baseline body mass index category - Indicators for adherence at 6m, 1y, and 2y |
| Outcomes | - Follow-up cardiometabolic parameters: Total cholesterol, LDL-C, HDL-C, triglycerides, TC:HDL ratio, body weight, systolic blood pressure, diastolic blood pressure, glucose, HbA1c (each all at 6m, 1y, and 2y) - Effectiveness outcomes: Mortality follow-up time (ITT), indicators for mortality by 6m, 1y, and 2y (ITT), mortality follow-up time (PP), indicator for died by 2y (PP), discontinuation follow-up time, indicator for discontinuation, indicator for switch to another study antipsychotic |

SMI, severe mental illness; LDL-C, low-density lipoprotein cholesterol; HDL-C, high-density lipoprotein cholesterol, TC:HDL, total cholesterol to high-density lipoprotein cholesterol; HbA1c, glycated haemoglobin; ITT, intention-to-treat; PP, per-protocol.

Variables were included in the imputation model as linear terms. Covariates were as specified in Table C.

Variables relating to psychiatric hospitalisation were not included as hospitalisation data were only available for patients with linked HES data and this outcome was not imputed.

Table E. Illustrative power calculation for primary outcome.*

|  | **N per group** | |
| --- | --- | --- |
| **Delta** | *For 80% power* | *For 90% power* |
| 0.25 | 393 | 526 |
| 0.20 | 614 | 822 |
| 0.15 | 1091 | 1460 |
| 0.10 | 2454 | 3285 |

* Assuming a standard deviation of 1.25[1] and a significance level of 0.05.

Table F. Characteristics of study cohort compared to patients prescribed a study antipsychotic during the study period but who did not meet eligibility criteria.

| **Characteristic** | **Excluded**  N = 169,109 | **Included** N = 26,537 | **Standardised difference  (95% CI)** |
| --- | --- | --- | --- |
| **Gender, No. (%)** |  |  | 0.16 (0.15, 0.18) |
| Female | 79,960 (47.3%) | 14,710 (55.4%) |  |
| Male | 89,136 (52.7%) | 11,827 (44.6%) |  |
| Unknown | 13 | 0 |  |
| **Ethnicity, No. (%)** |  |  | 0.09 (0.08, 0.11) |
| Asian | 10,269 (6.6%) | 1,807 (7.6%) |  |
| Black | 12,256 (7.9%) | 1,649 (6.9%) |  |
| Mixed | 4,196 (2.7%) | 387 (1.6%) |  |
| Other | 2,654 (1.7%) | 356 (1.5%) |  |
| White | 126,323 (81.1%) | 19,600 (82.4%) |  |
| Unknown | 13,411 | 2,738 |  |
| **IMD quintile, No. (%)** |  |  | 0.10 (0.09, 0.12) |
| 1 (Least deprived) | 15,998 (11.3%) | 2,963 (13.5%) |  |
| 2 | 20,085 (14.1%) | 3,482 (15.9%) |  |
| 3 | 25,794 (18.2%) | 4,153 (19.0%) |  |
| 4 | 35,727 (25.2%) | 5,131 (23.4%) |  |
| 5 (Most deprived) | 44,369 (31.3%) | 6,183 (28.2%) |  |
| Unknown | 27,136 | 4,625 |  |
| **Region, No. (%)** |  |  | 0.10 (0.09, 0.11) |
| East Midlands | 2,821 (1.7%) | 523 (2.0%) |  |
| East of England | 6,433 (3.8%) | 1,088 (4.1%) |  |
| London | 35,070 (20.7%) | 5,254 (19.8%) |  |
| North East | 4,120 (2.4%) | 802 (3.0%) |  |
| North West | 28,425 (16.8%) | 4,325 (16.3%) |  |
| Northern Ireland | 2,499 (1.5%) | 610 (2.3%) |  |
| Scotland | 12,117 (7.2%) | 1,973 (7.4%) |  |
| South East | 28,116 (16.6%) | 4,072 (15.3%) |  |
| South West | 16,588 (9.8%) | 2,292 (8.6%) |  |
| Wales | 9,078 (5.4%) | 1,477 (5.6%) |  |
| West Midlands | 20,024 (11.8%) | 3,418 (12.9%) |  |
| Yorkshire & The Humber | 3,818 (2.3%) | 703 (2.6%) |  |
| **Most recent SMI diagnosis, No. (%)** |  |  | 0.22 (0.20, 0.23) |
| Bipolar disorder | 57,435 (34.0%) | 11,264 (42.4%) |  |
| Other non-organic psychosis | 63,959 (37.8%) | 9,954 (37.5%) |  |
| Schizophrenia | 47,715 (28.2%) | 5,319 (20.0%) |  |
| **Age at first diagnosis (years), median (IQR)** | 33 (24, 46) | 43 (31, 58) | -0.47 (-0.48, -0.46) |
| **Age at first antipsychotic prescription, median (IQR)** | 38 (28, 51) | 50 (39, 63) | -0.55 (-0.56, -0.54) |
| **Antipsychotic initiation time-period, No. (%)** |  |  | 0.42 (0.41, 0.43) |
| <2005 | 12,220 (7.2%) | 5,511 (20.8%) |  |
| 2005-2009 | 56,117 (33.2%) | 6,295 (23.7%) |  |
| 2010-2014 | 65,732 (38.9%) | 9,086 (34.2%) |  |
| 2015+ | 35,040 (20.7%) | 5,645 (21.3%) |  |
| **Age at study start (years), median (IQR)** | 33 (22, 47) | 47 (35, 61) | -0.61 (-0.62, -0.59) |

IMD, index of multiple deprivation, SMI, severe mental illness.

Table G. Unadjusted cardiometabolic parameters at baseline and at 6m, 1y, and 2y follow-up.*

|  | **Intention-to-treat** | | | | **Per-protocol*^1^*** | | | |
| --- | --- | --- | --- | --- | --- | --- | --- | --- |
| **Time-point** | **Aripiprazole** | **Olanzapine** | **Quetiapine** | **Risperidone** | **Aripiprazole** | **Olanzapine** | **Quetiapine** | **Risperidone** |
| **Total cholesterol (mmol/L)** | | | | | | | | |
| Baseline | 4.94 (1.19) | 5.10 (1.18) | 5.07 (1.17) | 4.95 (1.16) | 4.94 (1.19) | 5.10 (1.18) | 5.07 (1.17) | 4.95 (1.16) |
| 6m | 4.87 (1.12) | 5.10 (1.16) | 5.01 (1.14) | 4.87 (1.14) | 4.86 (1.13) | 5.11 (1.15) | 5.03 (1.14) | 4.87 (1.14) |
| 1y | 4.92 (1.12) | 5.05 (1.14) | 5.04 (1.14) | 4.91 (1.14) | 4.90 (1.13) | 5.05 (1.15) | 5.03 (1.16) | 4.87 (1.14) |
| 2y | 4.84 (1.17) | 4.95 (1.16) | 4.93 (1.16) | 4.79 (1.16) | 4.86 (1.19) | 4.96 (1.14) | 4.96 (1.17) | 4.77 (1.13) |
| **LDL-C (mmol/L)** | | | | | | | | |
| Baseline | 2.82 (0.99) | 2.96 (1.01) | 2.91 (1.01) | 2.85 (1.01) | 2.82 (0.99) | 2.96 (1.01) | 2.91 (1.01) | 2.85 (1.01) |
| 6m | 2.74 (1.05) | 2.92 (1.07) | 2.83 (1.06) | 2.74 (1.05) | 2.72 (1.06) | 2.93 (1.07) | 2.83 (1.07) | 2.74 (1.05) |
| 1y | 2.76 (1.04) | 2.90 (1.05) | 2.86 (1.05) | 2.77 (1.04) | 2.73 (1.05) | 2.88 (1.06) | 2.84 (1.06) | 2.72 (1.04) |
| 2y | 2.65 (1.03) | 2.79 (1.02) | 2.74 (1.02) | 2.66 (1.02) | 2.66 (1.05) | 2.80 (1.02) | 2.76 (1.02) | 2.65 (1.00) |
| **HDL-C (mmol/L)** | | | | | | | | |
| Baseline | 1.36 (0.43) | 1.43 (0.44) | 1.40 (0.43) | 1.40 (0.45) | 1.36 (0.43) | 1.43 (0.44) | 1.40 (0.43) | 1.40 (0.45) |
| 6m | 1.34 (0.37) | 1.37 (0.39) | 1.36 (0.38) | 1.35 (0.38) | 1.34 (0.37) | 1.37 (0.39) | 1.36 (0.37) | 1.36 (0.38) |
| 1y | 1.34 (0.37) | 1.35 (0.38) | 1.35 (0.37) | 1.34 (0.38) | 1.33 (0.36) | 1.35 (0.38) | 1.35 (0.37) | 1.34 (0.39) |
| 2y | 1.34 (0.39) | 1.36 (0.39) | 1.35 (0.39) | 1.34 (0.39) | 1.33 (0.39) | 1.35 (0.39) | 1.34 (0.38) | 1.34 (0.39) |
| **Triglycerides (mmol/L)** | | | | | | | | |
| Baseline | 1.75 (1.27) | 1.60 (1.10) | 1.71 (1.27) | 1.60 (1.06) | 1.75 (1.27) | 1.60 (1.10) | 1.71 (1.27) | 1.60 (1.06) |
| 6m | 1.75 (1.18) | 1.81 (1.28) | 1.85 (1.30) | 1.72 (1.12) | 1.76 (1.18) | 1.81 (1.21) | 1.87 (1.34) | 1.72 (1.12) |
| 1y | 1.84 (1.23) | 1.81 (1.16) | 1.85 (1.25) | 1.77 (1.15) | 1.86 (1.27) | 1.82 (1.17) | 1.88 (1.28) | 1.77 (1.18) |
| 2y | 1.89 (1.25) | 1.79 (1.16) | 1.86 (1.23) | 1.74 (1.10) | 1.94 (1.35) | 1.80 (1.12) | 1.93 (1.29) | 1.74 (1.05) |
| **TC:HDL ratio** | | | | | | | | |
| Baseline | 3.94 (1.48) | 3.84 (1.39) | 3.90 (1.38) | 3.82 (1.40) | 3.94 (1.48) | 3.84 (1.39) | 3.90 (1.38) | 3.82 (1.40) |
| 6m | 3.87 (1.23) | 3.99 (1.32) | 3.92 (1.27) | 3.82 (1.20) | 3.86 (1.23) | 4.00 (1.33) | 3.94 (1.29) | 3.81 (1.18) |
| 1y | 3.93 (1.25) | 4.00 (1.30) | 3.96 (1.33) | 3.90 (1.23) | 3.93 (1.25) | 4.00 (1.36) | 3.97 (1.41) | 3.87 (1.23) |
| 2y | 3.90 (1.34) | 3.94 (1.32) | 3.93 (1.33) | 3.89 (1.32) | 3.90 (1.38) | 3.92 (1.32) | 3.95 (1.34) | 3.82 (1.28) |
| **Weight (kg)** | | | | | | | | |
| Baseline | 83.09 (22.80) | 75.19 (17.49) | 78.90 (19.52) | 77.54 (19.78) | 83.09 (22.80) | 75.19 (17.49) | 78.90 (19.52) | 77.54 (19.78) |
| 6m | 84.38 (23.44) | 78.02 (18.39) | 80.30 (20.34) | 78.68 (20.56) | 84.91 (23.81) | 78.32 (18.22) | 81.21 (20.40) | 79.37 (20.59) |
| 1y | 84.36 (23.14) | 78.44 (18.56) | 80.79 (20.51) | 78.98 (20.63) | 85.62 (23.79) | 78.88 (18.38) | 82.38 (20.58) | 80.10 (20.59) |
| 2y | 85.33 (23.67) | 78.47 (19.00) | 80.86 (20.96) | 79.15 (21.21) | 87.09 (23.77) | 79.42 (18.53) | 83.61 (20.91) | 81.53 (21.17) |
| **Systolic blood pressure (mm Hg)** | | | | | | | | |
| Baseline | 126.43 (16.27) | 129.40 (17.44) | 128.10 (16.62) | 129.74 (17.24) | 126.43 (16.27) | 129.40 (17.44) | 128.10 (16.62) | 129.74 (17.24) |
| 6m | 124.60 (15.56) | 126.48 (16.16) | 125.90 (16.06) | 126.73 (16.39) | 124.78 (15.57) | 126.70 (16.12) | 125.88 (15.90) | 126.63 (16.28) |
| 1y | 124.59 (15.57) | 126.92 (16.00) | 126.50 (15.92) | 127.00 (16.40) | 125.04 (15.40) | 127.34 (15.91) | 126.74 (15.81) | 127.27 (16.51) |
| 2y | 125.34 (15.44) | 126.70 (15.62) | 126.56 (15.61) | 126.67 (15.73) | 125.70 (15.17) | 127.42 (15.48) | 126.57 (15.25) | 127.04 (15.56) |
| **Diastolic blood pressure (mm Hg)** | | | | | | | | |
| Baseline | 77.06 (10.09) | 77.78 (10.48) | 77.56 (10.18) | 77.06 (10.34) | 77.06 (10.09) | 77.78 (10.48) | 77.56 (10.18) | 77.06 (10.34) |
| 6m | 75.96 (9.83) | 76.83 (10.02) | 76.44 (9.97) | 75.65 (10.11) | 75.94 (9.79) | 76.92 (9.98) | 76.47 (9.90) | 75.59 (10.05) |
| 1y | 76.05 (9.79) | 76.91 (9.99) | 76.85 (9.95) | 75.62 (10.11) | 76.09 (9.62) | 77.08 (9.94) | 77.15 (9.85) | 75.68 (10.14) |
| 2y | 75.71 (10.00) | 76.22 (9.96) | 76.19 (9.96) | 75.05 (10.09) | 76.26 (9.55) | 76.90 (9.59) | 76.69 (9.59) | 75.63 (9.56) |
| **HbA1c (mmol/mol)** | | | | | | | | |
| Baseline | 42.67 (14.92) | 39.27 (9.52) | 40.64 (11.96) | 42.43 (13.56) | 42.67 (14.92) | 39.27 (9.52) | 40.64 (11.96) | 42.43 (13.56) |
| 6m | 42.17 (14.12) | 40.29 (10.55) | 40.71 (11.89) | 42.49 (13.35) | 42.51 (14.21) | 40.51 (10.57) | 41.04 (12.14) | 42.91 (13.39) |
| 1y | 43.05 (14.53) | 40.48 (10.49) | 41.55 (12.25) | 42.94 (13.93) | 43.48 (14.73) | 40.60 (10.25) | 41.85 (12.38) | 43.44 (14.44) |
| 2y | 43.43 (15.28) | 40.19 (10.69) | 41.75 (12.85) | 42.73 (14.26) | 43.64 (15.13) | 39.89 (9.80) | 41.83 (12.79) | 42.94 (14.15) |
| **Glucose (mmol/L)** | | | | | | | | |
| Baseline | 6.17 (3.21) | 5.59 (2.08) | 5.79 (2.52) | 6.16 (3.00) | 6.17 (3.21) | 5.59 (2.08) | 5.79 (2.52) | 6.16 (3.00) |
| 6m | 6.29 (3.32) | 5.73 (2.36) | 5.92 (2.73) | 6.17 (3.10) | 6.33 (3.37) | 5.73 (2.28) | 5.95 (2.78) | 6.21 (3.13) |
| 1y | 6.30 (3.25) | 5.80 (2.36) | 5.99 (2.75) | 6.38 (3.24) | 6.31 (3.25) | 5.74 (2.23) | 5.98 (2.74) | 6.39 (3.31) |
| 2y | 6.49 (3.60) | 5.92 (2.63) | 6.08 (2.96) | 6.52 (3.53) | 6.41 (3.44) | 5.78 (2.31) | 6.01 (2.85) | 6.44 (3.50) |

mmol/L, millimoles per litre; LDL-C, low-density lipoprotein cholesterol; HDL-C, high-density lipoprotein cholesterol; TC:HDL, total cholesterol to high-density lipoprotein; HbA1c, glycated haemoglobin; kg, kilogram; mm Hg, millimetres of mercury; mmol/mol, millimoles per mole.

* Values are mean (SD). Missing values were replaced using multiple imputation. Descriptive statistics were pooled across imputed datasets according to Rubin’s rules.

^1^Values were only included if patient had not discontinued or switched to another study antipsychotic by the relevant time-point.

Table H. Proportion of patients with observed cardiometabolic parameter data.*

| **Outcome, No. (%)** | **Aripiprazole** N = 3,573 | **Olanzapine** N = 8,554 | **Quetiapine** N = 8,289 | **Risperidone** N = 6,121 |
| --- | --- | --- | --- | --- |
| **Total cholesterol** | | | | |
| At baseline | 3,333 (93%) | 8,074 (94%) | 7,777 (94%) | 5,842 (95%) |
| At 6m | 1,070 (30%) | 2,337 (28%) | 2,360 (29%) | 1,781 (30%) |
| At 1y | 1,075 (31%) | 2,328 (28%) | 2,259 (28%) | 1,749 (30%) |
| At 2y | 1,358 (38%) | 3,171 (38%) | 3,111 (38%) | 2,297 (39%) |
| Any time-point | 3,409 (97%) | 8,136 (97%) | 7,855 (97%) | 5,793 (98%) |
| **HDL-C** | | | | |
| At baseline | 3,033 (85%) | 6,917 (81%) | 6,766 (82%) | 4,998 (82%) |
| At 6m | 965 (27%) | 2,030 (24%) | 2,099 (26%) | 1,533 (26%) |
| At 1y | 993 (29%) | 2,051 (25%) | 2,011 (25%) | 1,541 (26%) |
| At 2y | 1,294 (37%) | 2,873 (34%) | 2,890 (36%) | 2,105 (35%) |
| Any time-point | 3,224 (91%) | 7,402 (88%) | 7,246 (89%) | 5,272 (89%) |
| **LDL-C** | | | | |
| At baseline | 2,562 (72%) | 5,894 (69%) | 5,659 (68%) | 4,258 (70%) |
| At 6m | 733 (21%) | 1,637 (20%) | 1,629 (20%) | 1,226 (21%) |
| At 1y | 767 (22%) | 1,629 (20%) | 1,600 (20%) | 1,241 (21%) |
| At 2y | 1,016 (29%) | 2,313 (28%) | 2,324 (29%) | 1,706 (29%) |
| Any time-point | 2,837 (80%) | 6,508 (78%) | 6,276 (77%) | 4,614 (78%) |
| **Triglycerides** | | | | |
| At baseline | 2,692 (75%) | 6,240 (73%) | 6,065 (73%) | 4,523 (74%) |
| At 6m | 787 (22%) | 1,755 (21%) | 1,754 (22%) | 1,312 (22%) |
| At 1y | 797 (23%) | 1,727 (21%) | 1,711 (21%) | 1,302 (22%) |
| At 2y | 1,053 (30%) | 2,401 (29%) | 2,444 (30%) | 1,771 (30%) |
| Any time-point | 2,925 (83%) | 6,760 (81%) | 6,570 (81%) | 4,817 (81%) |
| **TC:HDL ratio** | | | | |
| At baseline | 3,057 (86%) | 6,992 (82%) | 6,838 (82%) | 5,049 (82%) |
| At 6m | 970 (27%) | 2,073 (25%) | 2,128 (26%) | 1,550 (26%) |
| At 1y | 992 (29%) | 2,061 (25%) | 2,031 (25%) | 1,537 (26%) |
| At 2y | 1,292 (37%) | 2,877 (34%) | 2,907 (36%) | 2,094 (35%) |
| Any time-point | 3,237 (92%) | 7,449 (89%) | 7,293 (90%) | 5,305 (89%) |
| **Weight** | | | | |
| At baseline | 2,952 (83%) | 6,489 (76%) | 6,608 (80%) | 4,885 (80%) |
| At 6m | 1,258 (36%) | 2,633 (31%) | 2,690 (33%) | 1,973 (33%) |
| At 1y | 1,201 (35%) | 2,586 (31%) | 2,562 (32%) | 1,927 (33%) |
| At 2y | 1,402 (40%) | 3,385 (40%) | 3,331 (41%) | 2,353 (40%) |
| Any time-point | 3,269 (93%) | 7,496 (89%) | 7,387 (91%) | 5,372 (90%) |
| **Systolic blood pressure** | | | | |
| At baseline | 3,330 (93%) | 7,796 (91%) | 7,700 (93%) | 5,737 (94%) |
| At 6m | 1,430 (41%) | 3,301 (39%) | 3,362 (41%) | 2,448 (41%) |
| At 1y | 1,447 (42%) | 3,281 (40%) | 3,218 (40%) | 2,320 (40%) |
| At 2y | 1,437 (41%) | 3,458 (41%) | 3,344 (41%) | 2,365 (40%) |
| Any time-point | 3,459 (98%) | 8,125 (97%) | 7,966 (98%) | 5,817 (98%) |
| **Diastolic blood pressure** | | | | |
| At baseline | 3,329 (93%) | 7,793 (91%) | 7,700 (93%) | 5,736 (94%) |
| At 6m | 1,430 (41%) | 3,298 (39%) | 3,362 (41%) | 2,450 (41%) |
| At 1y | 1,447 (42%) | 3,282 (40%) | 3,216 (40%) | 2,320 (40%) |
| At 2y | 1,438 (41%) | 3,459 (41%) | 3,346 (41%) | 2,364 (40%) |
| Any time-point | 3,457 (98%) | 8,125 (97%) | 7,966 (98%) | 5,817 (98%) |
| **Hba1c** | | | | |
| At baseline | 1,878 (53%) | 2,855 (33%) | 3,175 (38%) | 2,575 (42%) |
| At 6m | 792 (22%) | 1,113 (13%) | 1,289 (16%) | 1,114 (19%) |
| At 1y | 822 (24%) | 1,207 (15%) | 1,294 (16%) | 1,157 (20%) |
| At 2y | 956 (27%) | 1,670 (20%) | 1,833 (23%) | 1,350 (23%) |
| Any time-point | 2,255 (64%) | 3,825 (46%) | 4,073 (50%) | 3,171 (53%) |
| **Glucose** | | | | |
| At baseline | 2,705 (76%) | 6,736 (79%) | 6,584 (79%) | 4,831 (79%) |
| At 6m | 740 (21%) | 1,941 (23%) | 1,866 (23%) | 1,417 (24%) |
| At 1y | 680 (20%) | 1,831 (22%) | 1,809 (23%) | 1,311 (23%) |
| At 2y | 869 (25%) | 2,431 (29%) | 2,316 (28%) | 1,652 (28%) |
| Any time-point | 2,927 (83%) | 7,186 (86%) | 7,008 (86%) | 5,106 (86%) |

LDL-C, low-density lipoprotein cholesterol; HDL-C, high-density lipoprotein cholesterol; TC:HDL, total cholesterol to high-density lipoprotein; HbA1c, glycated haemoglobin.

* Values were considered baseline if measured on the index date or within the two years prior (using the most recent value, where multiple values were available). Time-windows for cardiometabolic outcomes included a period before and after the study outcome time-point (e.g., at 6 ± 3 months, 12 ± 3 months, and 24 ± 6 months), with the measurement closest to the time-point used. Percentages are of patients alive at each time-point. Patients are counted in the denominator for the 'any time-point' measure if alive at six months post-index date.

Table I. Comparison of baseline characteristics of patients with and without observed primary outcome data.*

| **Characteristic** | **Missing** N = 18,142 | **Observed** N = 7,411 |
| --- | --- | --- |
| **Treatment group, No. (%)** |  |  |
| Aripiprazole | 2,405 (13.3%) | 1,075 (14.5%) |
| Olanzapine | 5,916 (32.6%) | 2,328 (31.4%) |
| Quetiapine | 5,754 (31.7%) | 2,259 (30.5%) |
| Risperidone | 4,067 (22.4%) | 1,749 (23.6%) |
| **Age at baseline (years), median (IQR)** | 51 (40, 65) | 56 (45, 68) |
| **Age at baseline (years), No. (%)** |  |  |
| Under 50 | 8,335 (45.9%) | 2,574 (34.7%) |
| 50 or over | 9,807 (54.1%) | 4,837 (65.3%) |
| **Sex, No. (%)** |  |  |
| Female | 10,069 (55.5%) | 4,103 (55.4%) |
| Male | 8,073 (44.5%) | 3,308 (44.6%) |
| **Ethnicity, No. (%)** |  |  |
| Asian | 1,206 (7.4%) | 579 (8.7%) |
| Black | 1,121 (6.9%) | 488 (7.3%) |
| Mixed | 269 (1.7%) | 112 (1.7%) |
| Other | 254 (1.6%) | 95 (1.4%) |
| White | 13,430 (82.5%) | 5,384 (80.9%) |
| Unknown | 1,862 | 753 |
| **Diagnosis, No. (%)** |  |  |
| Bipolar disorder | 7,482 (41.2%) | 3,203 (43.2%) |
| Other non-organic psychoses | 7,445 (41.0%) | 2,805 (37.8%) |
| Schizophrenia | 3,215 (17.7%) | 1,403 (18.9%) |
| **Age at diagnosis (years), median (IQR)** | 42 (30, 56) | 45 (33, 59) |
| **Prescribed an antipsychotic in last 2y, No. (%)** | 3,631 (20.0%) | 1,768 (23.9%) |
| **Psychiatric hospitalisation in last 2y, No. (%)** | 4,230 (28.8%) | 1,597 (26.8%) |
| Unknown | 3,473 | 1,461 |
| **Index date time-period, No. (%)** |  |  |
| 2005-2009 | 5,619 (31.0%) | 2,142 (28.9%) |
| 2010-2014 | 7,628 (42.0%) | 3,472 (46.8%) |
| 2015+ | 4,895 (27.0%) | 1,797 (24.2%) |
| **IMD quintile, No. (%)** |  |  |
| 1 (Least deprived) | 2,065 (13.8%) | 769 (12.7%) |
| 2 | 2,341 (15.6%) | 984 (16.2%) |
| 3 | 2,787 (18.6%) | 1,193 (19.7%) |
| 4 | 3,590 (23.9%) | 1,381 (22.8%) |
| 5 (Most deprived) | 4,232 (28.2%) | 1,743 (28.7%) |
| Unknown | 3,127 | 1,341 |
| **Comorbidities, No. (%)** |  |  |
| Alcohol misuse | 2,025 (11.2%) | 763 (10.3%) |
| Cerebrovascular disease | 1,041 (5.7%) | 567 (7.7%) |
| Diabetes | 3,011 (16.6%) | 2,160 (29.1%) |
| Dyslipidaemia | 3,211 (17.7%) | 1,995 (26.9%) |
| Hypertension | 4,898 (27.0%) | 2,772 (37.4%) |
| Liver disease | 406 (2.2%) | 157 (2.1%) |
| Myocardial infarction | 506 (2.8%) | 327 (4.4%) |
| Renal disease | 1,680 (9.3%) | 969 (13.1%) |
| Substance misuse | 1,621 (8.9%) | 440 (5.9%) |
| **Concomitant medications, No. (%)** |  |  |
| Antidepressant | 10,837 (59.7%) | 4,418 (59.6%) |
| Mood stabiliser | 5,165 (28.5%) | 2,275 (30.7%) |
| Lipid-regulating medication | 4,669 (25.7%) | 3,097 (41.8%) |
| Antidiabetic | 1,722 (9.5%) | 1,496 (20.2%) |
| Antihypertensive | 6,587 (36.3%) | 3,476 (46.9%) |
| **Total cholesterol (mmol/L), mean (SD)** | 5.06 (1.16) | 5.01 (1.20) |
| Unknown | 1,196 | 268 |
| **BMI (mg/m2), mean (SD)** | 27.9 (6.5) | 29.0 (6.5) |
| Unknown | 4,238 | 1,238 |
| **BMI category, No. (%)** |  |  |
| Underweight | 464 (3.3%) | 129 (2.1%) |
| Healthy | 4,572 (32.8%) | 1,654 (26.7%) |
| Overweight | 4,506 (32.3%) | 2,051 (33.2%) |
| Obese | 4,396 (31.5%) | 2,351 (38.0%) |
| Unknown | 4,204 | 1,226 |
| **Smoking status, No. (%)** |  |  |
| Never smoked | 7,058 (39.1%) | 3,082 (41.7%) |
| Ex-smoker | 2,949 (16.3%) | 1,316 (17.8%) |
| Current smoker | 8,037 (44.5%) | 2,999 (40.5%) |
| Unknown | 98 | 14 |
| **Number of primary care consults in last 6m, median (IQR)** | 6 (3, 10) | 7 (4, 11) |
| **Starting daily dose (Olanzapine equivalent, mg), mean (SD)** | 6.0 (5.2) | 5.7 (4.9) |
| Unknown | 3,291 | 1,342 |

IMD, index of multiple deprivation; BMI, body mass index; mg, milligram.

The primary outcome was the total cholesterol level at one year.

* Amongst patients alive at one-year post-index date.

Table J. Cardiometabolic outcomes with aripiprazole versus comparator antipsychotics – intention-to-treat and per-protocol results.*

|  | **Intention-to-treat*^1^*** |  |  | **Per-protocol*^2^*** |  |  |
| --- | --- | --- | --- | --- | --- | --- |
|  | Aripiprazole vs. Olanzapine | Aripiprazole vs. Quetiapine | Aripiprazole vs. Risperidone | Aripiprazole vs. Olanzapine | Aripiprazole vs. Quetiapine | Aripiprazole vs. Risperidone |
| **Total cholesterol (mmol/L)** |  |  |  |  |  |  |
| 6m | -0.12 (-0.19, -0.04) | -0.04 (-0.11, 0.02) | -0.03 (-0.12, 0.06) | -0.13 (-0.21, -0.04) | -0.05 (-0.12, 0.02) | -0.03 (-0.13, 0.07) |
| 1y | -0.03 (-0.09, 0.02) | -0.03 (-0.09, 0.03) | -0.01 (-0.08, 0.05) | -0.04 (-0.11, 0.03) | -0.03 (-0.11, 0.04) | 0.00 (-0.08, 0.08) |
| 2y | 0.01 (-0.05, 0.06) | 0.01 (-0.04, 0.06) | 0.03 (-0.03, 0.09) | 0.03 (-0.06, 0.11) | 0.02 (-0.05, 0.10) | 0.07 (-0.01, 0.16) |
| **LDL-C (mmol/L)** |  |  |  |  |  |  |
| 6m | -0.08 (-0.16, -0.01) | -0.01 (-0.08, 0.06) | -0.02 (-0.11, 0.07) | -0.10 (-0.18, -0.02) | -0.02 (-0.09, 0.05) | -0.02 (-0.12, 0.08) |
| 1y | -0.04 (-0.11, 0.03) | -0.03 (-0.10, 0.04) | -0.02 (-0.10, 0.05) | -0.04 (-0.11, 0.02) | -0.03 (-0.10, 0.05) | -0.01 (-0.10, 0.07) |
| 2y | -0.04 (-0.10, 0.02) | -0.02 (-0.07, 0.03) | -0.02 (-0.08, 0.04) | -0.02 (-0.10, 0.06) | 0.00 (-0.08, 0.08) | 0.01 (-0.08, 0.09) |
| **HDL-C (mmol/L)** |  |  |  |  |  |  |
| 6m | 0.02 (-0.01, 0.06) | 0.02 (-0.01, 0.05) | 0.01 (-0.02, 0.04) | 0.03 (0.00, 0.06) | 0.02 (-0.01, 0.05) | 0.02 (-0.02, 0.05) |
| 1y | 0.03 (0.01, 0.05) | 0.01 (-0.01, 0.03) | 0.02 (-0.01, 0.04) | 0.03 (0.01, 0.06) | 0.01 (-0.01, 0.03) | 0.01 (-0.01, 0.04) |
| 2y | 0.03 (0.01, 0.05) | 0.02 (0.00, 0.04) | 0.03 (0.01, 0.05) | 0.04 (0.01, 0.06) | 0.02 (0.00, 0.05) | 0.03 (0.00, 0.05) |
| **Triglycerides (mmol/L)** |  |  |  |  |  |  |
| 6m | -0.14 (-0.23, -0.05) | -0.12 (-0.20, -0.04) | -0.06 (-0.16, 0.04) | -0.14 (-0.24, -0.04) | -0.13 (-0.22, -0.04) | -0.06 (-0.16, 0.05) |
| 1y | -0.07 (-0.14, 0.01) | -0.04 (-0.11, 0.03) | -0.01 (-0.09, 0.07) | -0.08 (-0.17, 0.02) | -0.05 (-0.13, 0.03) | -0.01 (-0.10, 0.09) |
| 2y | 0.02 (-0.06, 0.09) | 0.01 (-0.06, 0.09) | 0.05 (-0.04, 0.14) | 0.02 (-0.07, 0.12) | 0.00 (-0.10, 0.10) | 0.09 (-0.02, 0.20) |
| **TC:HDL ratio** |  |  |  |  |  |  |
| 6m | -0.16 (-0.25, -0.07) | -0.06 (-0.15, 0.03) | -0.05 (-0.15, 0.04) | -0.19 (-0.29, -0.09) | -0.08 (-0.18, 0.02) | -0.06 (-0.17, 0.05) |
| 1y | -0.11 (-0.18, -0.04) | -0.04 (-0.11, 0.03) | -0.06 (-0.15, 0.02) | -0.14 (-0.22, -0.06) | -0.06 (-0.14, 0.03) | -0.06 (-0.15, 0.03) |
| 2y | -0.07 (-0.14, 0.00) | -0.03 (-0.10, 0.04) | -0.06 (-0.13, 0.01) | -0.07 (-0.17, 0.03) | -0.03 (-0.13, 0.06) | -0.03 (-0.13, 0.07) |
| **Weight (kg)** |  |  |  |  |  |  |
| 6m | -1.03 (-1.52, -0.54) | 0.10 (-0.46, 0.66) | -0.25 (-0.87, 0.37) | -1.20 (-1.74, -0.66) | 0.04 (-0.56, 0.63) | -0.32 (-0.97, 0.33) |
| 1y | -1.50 (-2.10, -0.91) | -0.41 (-0.98, 0.16) | -0.76 (-1.41, -0.11) | -1.68 (-2.34, -1.02) | -0.47 (-1.12, 0.17) | -0.79 (-1.50, -0.08) |
| 2y | -0.39 (-1.10, 0.32) | 0.54 (-0.20, 1.28) | 0.06 (-0.64, 0.76) | -0.68 (-1.58, 0.22) | 0.38 (-0.57, 1.33) | -0.07 (-0.99, 0.85) |
| **Systolic blood pressure (mm Hg)** |  |  |  |  |  |  |
| 6m | -0.90 (-1.83, 0.04) | -0.86 (-1.86, 0.14) | -0.30 (-1.20, 0.61) | -1.01 (-2.00, -0.02) | -0.95 (-2.06, 0.16) | -0.04 (-1.05, 0.98) |
| 1y | -1.04 (-2.01, -0.08) | -1.14 (-2.06, -0.22) | -0.44 (-1.55, 0.68) | -1.19 (-2.26, -0.13) | -1.25 (-2.32, -0.18) | -0.38 (-1.64, 0.88) |
| 2y | -0.28 (-1.23, 0.67) | -0.66 (-1.48, 0.17) | 0.42 (-0.47, 1.32) | -0.85 (-2.11, 0.42) | -0.71 (-1.85, 0.44) | 0.23 (-0.99, 1.45) |
| **Diastolic blood pressure (mm Hg)** |  |  |  |  |  |  |
| 6m | -1.10 (-1.67, -0.54) | -0.55 (-1.16, 0.05) | -0.33 (-0.88, 0.21) | -1.17 (-1.78, -0.56) | -0.50 (-1.15, 0.16) | -0.25 (-0.86, 0.36) |
| 1y | -0.92 (-1.46, -0.38) | -0.80 (-1.39, -0.21) | -0.05 (-0.76, 0.65) | -1.16 (-1.80, -0.52) | -0.99 (-1.67, -0.31) | -0.17 (-1.00, 0.67) |
| 2y | -0.78 (-1.37, -0.20) | -0.68 (-1.27, -0.08) | -0.21 (-0.79, 0.36) | -1.03 (-1.75, -0.31) | -0.45 (-1.19, 0.28) | -0.18 (-0.93, 0.57) |
| **HbA1c (mmol/mol)** |  |  |  |  |  |  |
| 6m | -1.16 (-2.07, -0.25) | -0.48 (-1.36, 0.39) | -0.71 (-1.56, 0.13) | -1.29 (-2.28, -0.30) | -0.57 (-1.50, 0.37) | -0.78 (-1.70, 0.14) |
| 1y | -0.20 (-1.15, 0.75) | -0.11 (-0.92, 0.71) | 0.07 (-0.75, 0.89) | -0.33 (-1.33, 0.68) | -0.09 (-1.01, 0.82) | -0.06 (-0.99, 0.86) |
| 2y | 0.40 (-0.26, 1.05) | -0.03 (-0.74, 0.67) | 0.64 (-0.04, 1.32) | 0.35 (-0.57, 1.27) | -0.06 (-0.99, 0.88) | 0.67 (-0.25, 1.59) |
| **Glucose (mmol/L)** |  |  |  |  |  |  |
| 6m | -0.03 (-0.22, 0.15) | 0.02 (-0.15, 0.18) | 0.03 (-0.15, 0.22) | -0.05 (-0.25, 0.14) | 0.00 (-0.18, 0.18) | 0.01 (-0.18, 0.20) |
| 1y | -0.11 (-0.23, 0.01) | -0.05 (-0.18, 0.08) | -0.13 (-0.28, 0.02) | -0.14 (-0.29, 0.01) | -0.06 (-0.22, 0.11) | -0.19 (-0.36, -0.02) |
| 2y | -0.02 (-0.14, 0.10) | 0.08 (-0.04, 0.20) | -0.05 (-0.21, 0.10) | -0.07 (-0.26, 0.12) | 0.04 (-0.16, 0.24) | -0.11 (-0.32, 0.11) |

mmol/L, millimoles per litre; LDL-C, low-density lipoprotein cholesterol; HDL-C, high-density lipoprotein cholesterol; TC:HDL, total cholesterol to high-density lipoprotein; kg, kilogram; mm Hg, millimetres of mercury; mmol/mol, millimoles per mole.

Values are adjusted mean difference (95% confidence interval). Models were adjusted for age, sex, ethnicity, SMI diagnosis category, prior use of antipsychotics, level of deprivation (quintile), geographic region, calendar year of index date, number of primary care consultations in prior six months, smoking status, comorbidities (dyslipidaemia, diabetes, hypertension, cerebrovascular disease, myocardial infarction, renal disease, liver disease, alcohol misuse, substance misuse), concomitant medications (lipid-regulating medications, antihypertensives, antidiabetics, antidepressants, mood stabilisers) and cardiometabolic values (total cholesterol, LDL-C, HDL-C, triglycerides, systolic blood pressure, diastolic blood pressure, glucose, HbA1c, weight, BMI category). Missing values were replaced using multiple imputation.

^1^ Intention-to-treat results reported for patients alive at each time point. At 6m, 1y and 2y, denominators for Aripiprazole were: 3530, 3480, 3391; Olanzapine: 8383, 8244, 8003; Quetiapine: 8137, 8013, 7809; and Risperidone: 5941, 5816, 5548, respectively.

^2^ Per-protocol results reported for patients alive and who had not switched/discontinued at each time point, denominators for Aripiprazole were: 2619, 2026, 1427; Olanzapine: 6466, 4946, 3325; Quetiapine: 6300, 4858, 3436; and Risperidone: 4396, 3295, 2166, respectively.

Table K. Additional sensitivity analyses for the main outcomes.

|  | **Intention-to-treat** | **Per-protocol** |
| --- | --- | --- |
| **Total cholesterol at 1y (mmol/L)*^1^*** |  |  |
| *Accounting for clustering at the primary care practice level^2^* |  |  |
| Aripiprazole vs. Olanzapine | -0.03 (-0.09, 0.02) | -0.04 (-0.11, 0.03) |
| Aripiprazole vs. Quetiapine | -0.03 (-0.09, 0.03) | -0.03 (-0.11, 0.04) |
| Aripiprazole vs. Risperidone | -0.01 (-0.08, 0.05) | 0.00 (-0.08, 0.08) |
| *Inverse probability of treatment weighting^3,4^* |  |  |
| Aripiprazole vs. Olanzapine | -0.04 (-0.10, 0.02) | -0.01 (-0.11, 0.08) |
| Aripiprazole vs. Quetiapine | -0.04 (-0.11, 0.02) | -0.01 (-0.12, 0.09) |
| Aripiprazole vs. Risperidone | -0.03 (-0.11, 0.04) | -0.03 (-0.14, 0.08) |
| *Inverse probability of censoring weighting^5^* |  |  |
| Aripiprazole vs. Olanzapine | - | -0.03 (-0.11, 0.04) |
| Aripiprazole vs. Quetiapine | - | -0.03 (-0.11, 0.05) |
| Aripiprazole vs. Risperidone | - | 0.01 (-0.08, 0.09) |
| **Psychiatric hospitalisation*^1^*** |  |  |
| *More restricted definition^6,7^* |  |  |
| Aripiprazole vs. Olanzapine | 0.93 (0.83, 1.04) | - |
| Aripiprazole vs. Quetiapine | 0.99 (0.88, 1.11) | - |
| Aripiprazole vs. Risperidone | 1.08 (0.96, 1.22) | - |
| *Accounting for clustering at the primary care practice level^2,7^* |  |  |
| Aripiprazole vs. Olanzapine | 0.91 (0.83, 1.01) | 0.90 (0.80, 1.02) |
| Aripiprazole vs. Quetiapine | 0.94 (0.84, 1.04) | 0.90 (0.79, 1.01) |
| Aripiprazole vs. Risperidone | 1.01 (0.90, 1.13) | 0.95 (0.84, 1.07) |
| *Inverse probability of treatment weighting^3,8^* |  |  |
| Aripiprazole vs. Olanzapine | 0.90 (0.80, 1.01) | 0.86 (0.75, 0.99) |
| Aripiprazole vs. Quetiapine | 0.88 (0.78, 1.00) | 0.79 (0.69, 0.91) |
| Aripiprazole vs. Risperidone | 0.93 (0.82, 1.05) | 0.83 (0.72, 0.96) |
| *Inverse probability of censoring weighting^4^* |  |  |
| Aripiprazole vs. Olanzapine | - | 0.93 (0.82, 1.05) |
| Aripiprazole vs. Quetiapine | - | 0.91 (0.81, 1.04) |
| Aripiprazole vs. Risperidone | - | 0.98 (0.86, 1.12) |

^1^Total cholesterol at one year results were adjusted for all pre-specified covariates. Psychiatric hospitalisation results were adjusted for all pre-specified covariates plus prior psychiatric hospitalisation. See Table C for details of covariates.

^2^ Estimates calculated using robust standard errors which account for clustering at the primary care practice level.

^3^ Inverse probability of treatment weighting was successful in achieving covariate balance in the pseudo-population, with all pre-specified baseline covariates demonstrating <10% standardised mean differences for each comparator group relative to the aripiprazole group following weighting. See: Table C for details of covariates; Figure B for standardised differences; and Figure C for the distribution of the weights.

^4^ Estimates are weighted mean differences (95% CI).

^5^ Stabilised censoring weights were derived from a logistic model incorporating available baseline covariates (see Table C) and follow-up variables (i.e. indicators of missing data and cardiometabolic values over follow-up time-points). Weights were computed in each imputed dataset, with trimming at the 99.5^th^ percentile. Mean (SD) censoring weights across imputed datasets for the total cholesterol results, which accounted for censoring by one-year post-index date, were: Aripiprazole - 1.46 (0.33); Olanzapine - 1.56 (0.34); Quetiapine - 1.61 (0.35); and Risperidone - 1.36 (0.29). Mean (SD) censoring weights for the psychiatric hospitalisation results, which accounted for censoring by two years post-index date, were: Aripiprazole - 1.04 (0.23); Olanzapine - 1.04 (0.22), Quetiapine - 1.04 (0.23); and Risperidone - 1.03 (0.21).

^6^ The definition of psychiatric hospitalisation was further restricted by only including hospitalisations recorded in Hospital Episode Statistics data that also had a corresponding psychiatric consultant main or treatment specialty code for any episode of care within the hospitalisation (i.e., a psychiatrist was recorded as responsible for the care of the patient).

^7^ Estimates are adjusted cause-specific hazard ratios (95% CI).

^8^ Estimates are weighted cause-specific hazard ratios (95% CI).

Table L. Lipid-regulating medication prescriptions after index date.*

|  | **Aripiprazole** | **Olanzapine** | **Quetiapine** | **Risperidone** |
| --- | --- | --- | --- | --- |
| **Overall** | N = 3,480 | N = 8,244 | N = 8,013 | N = 5,816 |
| At baseline | 1,114 (32.0%) | 2,150 (26.1%) | 2,417 (30.2%) | 2,085 (35.8%) |
| Within 1y of index date | 1,118 (32.1%) | 2,152 (26.1%) | 2,380 (29.7%) | 2,076 (35.7%) |
| **No lipid-regulating medications at baseline** | N = 2,366 | N = 6,094 | N = 5,596 | N = 3,731 |
| Within 1y of index date | 105 (4.4%) | 269 (4.4%) | 233 (4.2%) | 205 (5.5%) |

** Among patients alive at one-year post-index date.*

Table M. Unadjusted estimated probabilities of each effectiveness outcome.

|  | **Time-point** |  |  |
| --- | --- | --- | --- |
|  | **6m** | **1y** | **2y** |
| **Psychiatric hospitalisation^1^** |  |  |  |
| Aripiprazole | 8.94% (7.94%, 10.0%) | 13.3% (12.1%, 14.6%) | 19.0% (17.6%, 20.4%) |
| Olanzapine | 10.8% (10.0%, 11.5%) | 16.1% (15.2%, 17.0%) | 22.1% (21.1%, 23.1%) |
| Quetiapine | 10.2% (9.46%, 10.9%) | 14.1% (13.2%, 14.9%) | 19.3% (18.3%, 20.2%) |
| Risperidone | 9.94% (9.14%, 10.8%) | 13.9% (12.9%, 14.9%) | 19.1% (18.0%, 20.1%) |
| **Discontinuation^1^** |  |  |  |
| Aripiprazole | 14.4% (13.2%, 15.6%) | 24.9% (23.4%, 26.3%) | 36.7% (35.0%, 38.3%) |
| Olanzapine | 12.7% (12.0%, 13.5%) | 23.5% (22.6%, 24.5%) | 38.0% (37.0%, 39.1%) |
| Quetiapine | 12.5% (11.8%, 13.2%) | 23.6% (22.7%, 24.6%) | 36.7% (35.6%, 37.8%) |
| Risperidone | 14.7% (13.9%, 15.7%) | 25.2% (24.1%, 26.3%) | 38.0% (36.7%, 39.2%) |
| **Mortality^2^** |  |  |  |
| Aripiprazole | 1.21% (0.85%, 1.57%) | 2.64% (2.11%, 3.16%) | 5.20% (4.46%, 5.94%) |
| Olanzapine | 2.01% (1.71%, 2.31%) | 3.65% (3.25%, 4.05%) | 6.54% (6.01%, 7.06%) |
| Quetiapine | 1.84% (1.55%, 2.13%) | 3.35% (2.96%, 3.74%) | 5.88% (5.37%, 6.39%) |
| Risperidone | 2.96% (2.53%, 3.38%) | 5.03% (4.47%, 5.57%) | 9.51% (8.76%, 10.2%) |

^1^ Estimated from the cumulative incidence function accounting for death as a potential competing risk.

^2^ Estimated from the survival function using the Kaplan-Meier method.

Table N. Psychiatric hospitalisation outcome with aripiprazole versus comparator antipsychotics – intention-to-treat and per-protocol results.

|  | **Cause-specific hazards model*^1^*** | | | **Subdistribution hazards model*^2^*** | | |
| --- | --- | --- | --- | --- | --- | --- |
| Comparison | Unadjusted | Model 1*^3^* | Model 2*^4^* | Unadjusted | Model 1*^3^* | Model 2*^4^* |
| **Intention-to-treat** |  |  |  |  |  |  |
| Aripiprazole vs. Olanzapine | 0.83 (0.76, 0.92) | 0.86 (0.77, 0.95) | 0.91 (0.82, 1.01) | 0.84 (0.76, 0.92) | 0.86 (0.77, 0.95) | 0.91 (0.83, 1.01) |
| Aripiprazole vs. Quetiapine | 0.97 (0.88, 1.07) | 0.98 (0.88, 1.08) | 0.94 (0.85, 1.04) | 0.97 (0.88, 1.08) | 0.98 (0.88, 1.09) | 0.94 (0.85, 1.04) |
| Aripiprazole vs. Risperidone | 0.97 (0.87, 1.08) | 1.02 (0.92, 1.14) | 1.01 (0.91, 1.12) | 0.99 (0.89, 1.09) | 1.02 (0.92, 1.14) | 1.02 (0.91, 1.13) |
| **Per-protocol^5^** |  |  |  |  |  |  |
| Aripiprazole vs. Olanzapine | 0.82 (0.73, 0.92) | 0.86 (0.77, 0.97) | 0.90 (0.80, 1.02) | 0.84 (0.75, 0.93) | 0.86 (0.76, 0.96) | 0.90 (0.80, 1.01) |
| Aripiprazole vs. Quetiapine | 0.92 (0.82, 1.03) | 0.93 (0.82, 1.05) | 0.89 (0.79, 1.01) | 0.92 (0.82, 1.03) | 0.93 (0.83, 1.05) | 0.90 (0.80, 1.01) |
| Aripiprazole vs. Risperidone | 0.90 (0.80, 1.02) | 0.96 (0.85, 1.09) | 0.95 (0.84, 1.08) | 0.95 (0.84, 1.07) | 0.98 (0.86, 1.11) | 0.97 (0.86, 1.10) |

Estimates are hazard ratios (95% confidence interval) for the effect of aripiprazole versus comparator antipsychotics.

^1^ Death was treated as a censoring event. Estimates are cause-specific hazard ratios.

^2^ Death was accounted for as a potential competing risk using the Fine-Gray subdistribution hazards model. Estimates are subdistribution hazard ratios.

^3^ Adjusted for pre-specified baseline covariates: age, sex, ethnicity, SMI diagnosis category, prior use of antipsychotics, level of deprivation (quintile), geographic region, calendar year of index date, number of primary care consultations in prior six months, smoking status, comorbidities (dyslipidaemia, diabetes, hypertension, cerebrovascular disease, myocardial infarction, renal disease, liver disease, alcohol misuse, substance misuse), concomitant medications (lipid-regulating medications, antihypertensives, antidiabetics, antidepressants, mood stabilisers) and cardiometabolic values (total cholesterol, LDL-C, HDL-C, triglycerides, systolic blood pressure, diastolic blood pressure, glucose, HbA1c, weight, BMI category).

^4^ As above, but additionally adjusted for psychiatric hospitalization in the prior two years.

^5^ Patients were censored at discontinuation or switch to one of the other study medications.

Table O. Discontinuation and mortality outcomes with aripiprazole versus comparator antipsychotics – intention-to-treat and per-protocol results.

|  | **Intention-to-treat** |  |  |  | **Per-protocol*^1^*** |  |
| --- | --- | --- | --- | --- | --- | --- |
|  | **Cause-specific hazards model*^2^*** |  | **Subdistribution hazards model*^3^*** |  | **Cause-specific hazards model*^2^*** |  |
|  | Unadjusted | Adjusted*^4^* | Unadjusted | Adjusted*^4^* | Unadjusted | Adjusted*^4^* |
| **Discontinuation** |  |  |  |  |  |  |
| Aripiprazole vs. Olanzapine | 0.97 (0.91, 1.04) | 1.04 (0.97, 1.11) | 0.98 (0.91, 1.04) | 1.03 (0.96, 1.10) | - | - |
| Aripiprazole vs. Quetiapine | 1.01 (0.95, 1.08) | 1.01 (0.95, 1.09) | 1.02 (0.95, 1.08) | 1.01 (0.94, 1.08) | - | - |
| Aripiprazole vs. Risperidone | 0.94 (0.88, 1.01) | 0.98 (0.92, 1.06) | 0.97 (0.90, 1.03) | 0.99 (0.92, 1.06) | - | - |
| **Mortality** |  |  |  |  |  |  |
| Aripiprazole vs. Olanzapine | 0.79 (0.67, 0.93) | 0.94 (0.79, 1.11) | - | - | 0.73 (0.59, 0.91) | 0.85 (0.68, 1.07) |
| Aripiprazole vs. Quetiapine | 0.88 (0.74, 1.04) | 1.05 (0.88, 1.26) | - | - | 0.90 (0.72, 1.12) | 0.97 (0.77, 1.22) |
| Aripiprazole vs. Risperidone | 0.53 (0.45, 0.63) | 0.88 (0.74, 1.05) | - | - | 0.53 (0.42, 0.65) | 0.84 (0.67, 1.05) |

Estimates are hazard ratios (95% confidence interval) for the effect of aripiprazole versus comparator antipsychotics.

^1^ Patients were censored at discontinuation or switch to one of the other study medications.

^2^ For discontinuation, death was treated as a censoring event. Estimates are cause-specific hazard ratios.

^3^ Death was accounted for as a potential competing risk using the Fine-Gray subdistribution hazards model. Estimates are subdistribution hazard ratios.

^4^ Adjusted for pre-specified baseline covariates: age, sex, ethnicity, SMI diagnosis category, prior use of antipsychotics, level of deprivation (quintile), geographic region, calendar year of index date, number of primary care consultations in prior six months, smoking status, comorbidities (dyslipidaemia, diabetes, hypertension, cerebrovascular disease, myocardial infarction, renal disease, liver disease, alcohol misuse, substance misuse), concomitant medications (lipid-regulating medications, antihypertensives, antidiabetics, antidepressants, mood stabilisers) and cardiometabolic values (total cholesterol, LDL-C, HDL-C, triglycerides, systolic blood pressure, diastolic blood pressure, glucose, HbA1c, weight, BMI category).


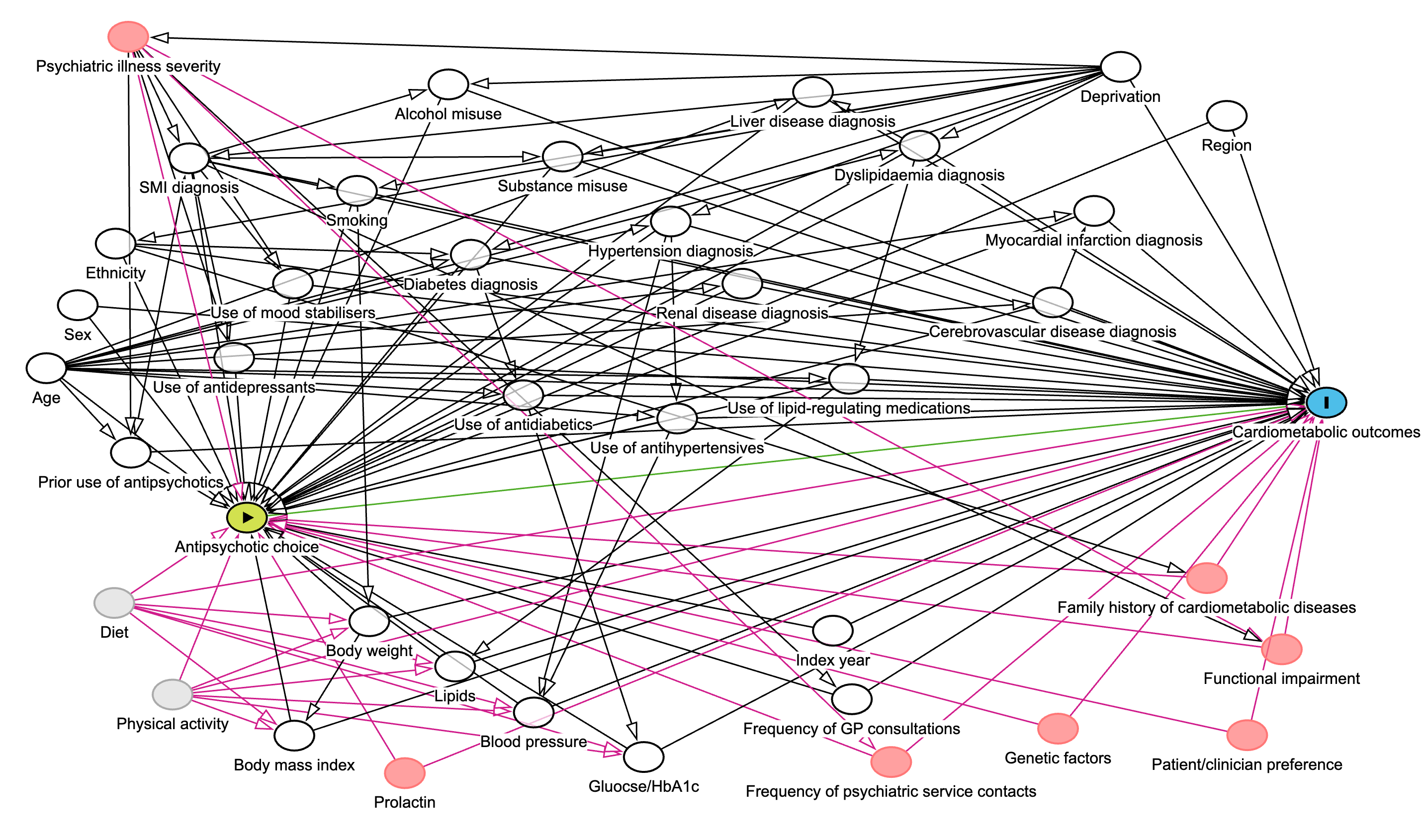


Figure A. Directed acyclic graph for the baseline confounding structure for cardiometabolic safety outcomes.

Although unmeasured confounders were identified, we concluded that the measured confounders likely captured the majority of the confounding, ensuring robust results. We deemed that a similar confounder structure was plausible for the effectiveness outcomes and used the same confounder set in all adjusted analyses for consistency (note that we added prior psychiatric hospitalisation post-hoc when investigating the hospitalisation outcome).


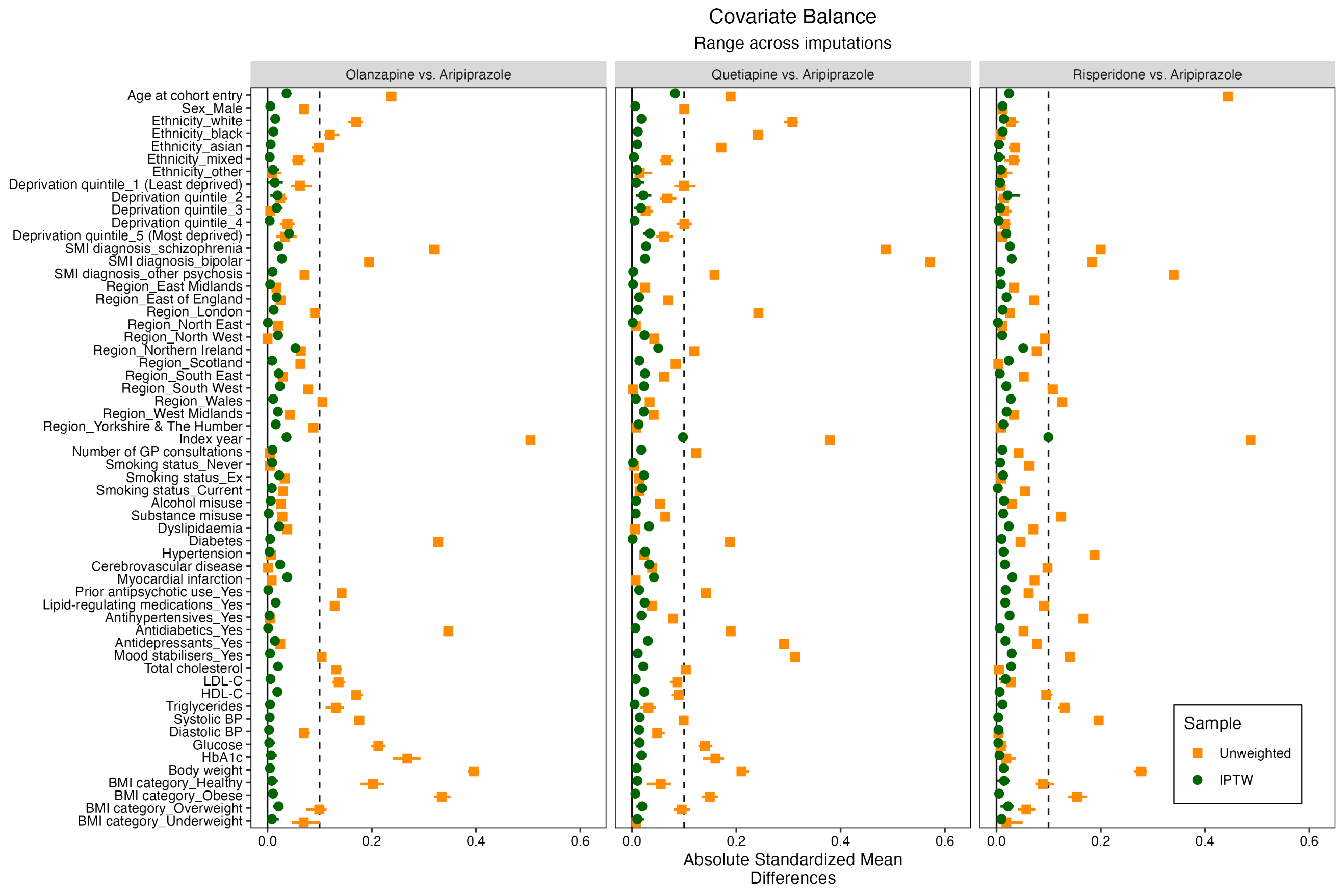


Figure B. Inverse probability of treatment weighting sensitivity analysis - covariate balance plot.

Standardised mean differences for covariates in the unweighted and inverse probability of treatment weighted comparisons of aripiprazole versus each comparator across imputed datasets; all demonstrated <10% differences for each comparison after weighting. Weighting and balance diagnostics performed using MatchThem and cobalt.[2], [3]


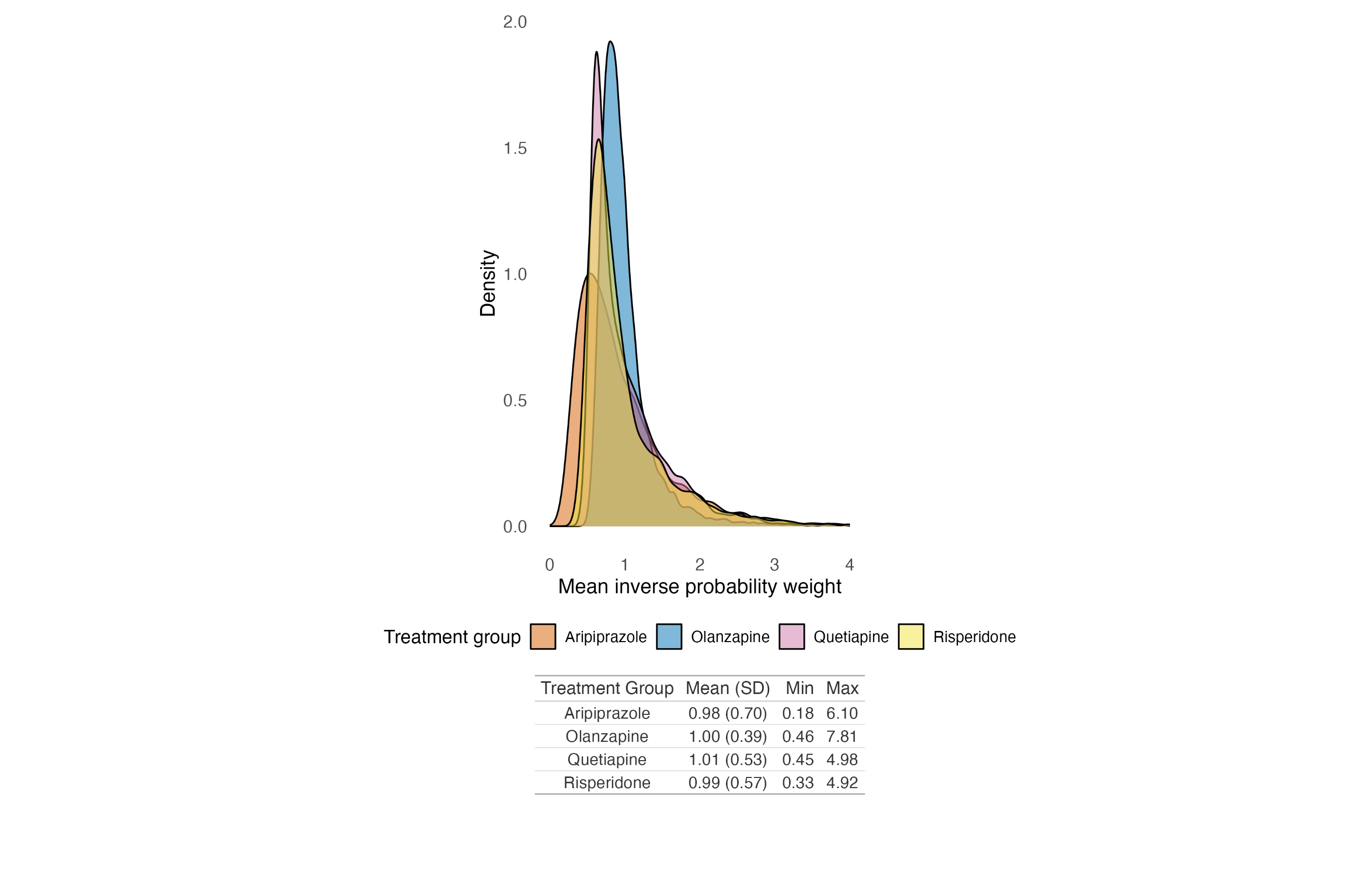


Figure C. Inverse probability of treatment weighting sensitivity analysis - distribution of weights.

Density plot and descriptive statistics of inverse probability weights estimated across imputed datasets.


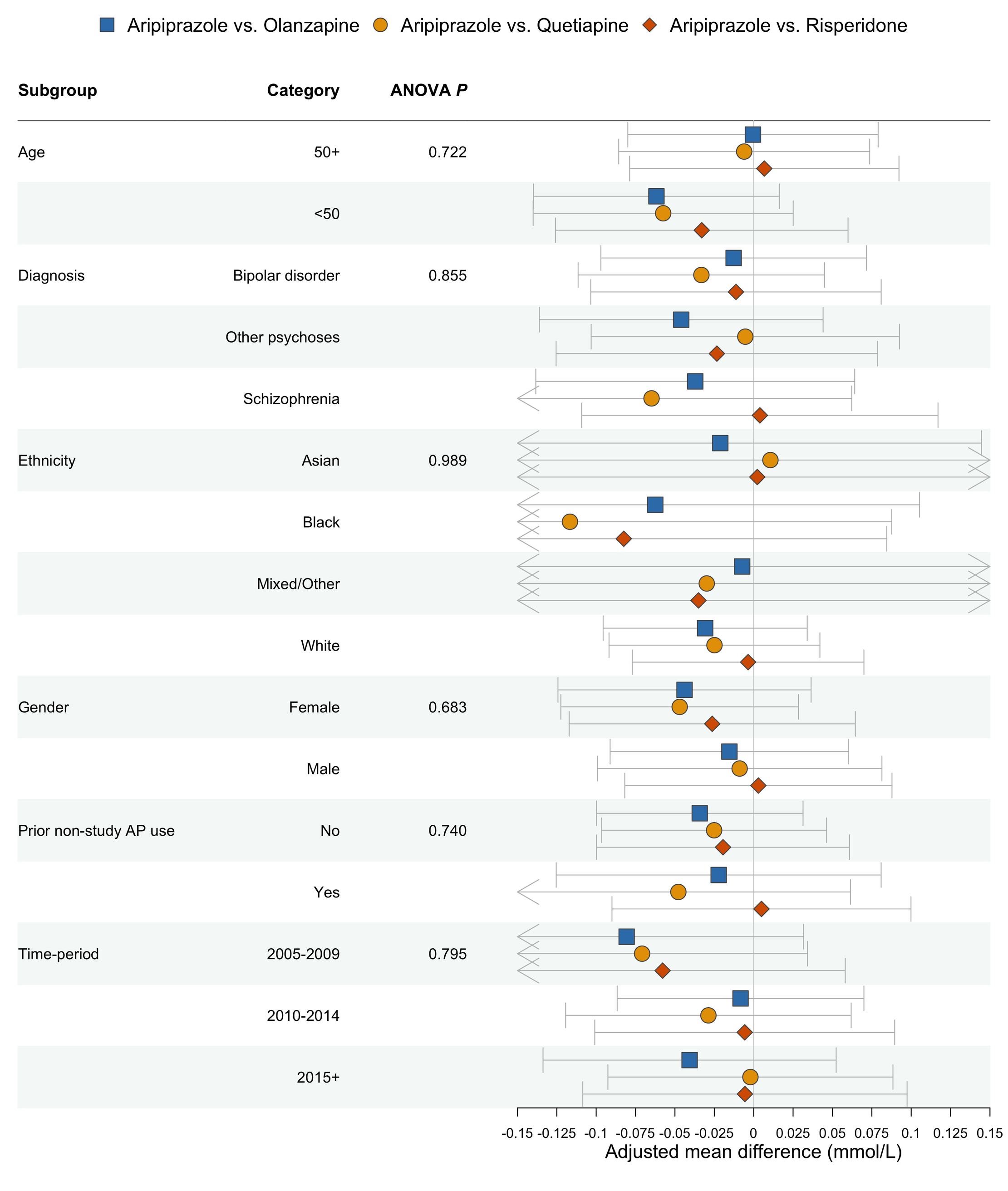


Figure D. Total cholesterol at one year outcome across subgroups.

AP, antipsychotic; mmol/L, millimoles per litre.

The forest plot shows the adjusted mean difference (mmol/L) of aripiprazole versus each comparator antipsychotic in each subgroup from the intention-to-treat analysis. Estimates to the left of the no effect line (at mean difference = 0) favour aripiprazole whilst those to the right favour a comparator. Models were adjusted for pre-specified covariates of: age, sex, ethnicity, SMI diagnosis category, prior use of antipsychotics, level of deprivation (quintile), geographic region, calendar year of index date, number of primary care consultations in prior six months, smoking status, comorbidities (dyslipidaemia, diabetes, hypertension, cerebrovascular disease, myocardial infarction, renal failure, liver disease, alcohol misuse, substance misuse), concomitant medications (lipid-regulating medications, antihypertensives, antidiabetics, antidepressants, mood stabilisers) and cardiometabolic values (total cholesterol, LDL-C, HDL-C, triglycerides, systolic blood pressure, diastolic blood pressure, glucose, HbA1c, weight, BMI category).

Supporting References

[1] A. Richards-Belle *et al.*, ‘Associations of antidepressants and antipsychotics with lipid parameters: Do CYP2C19/CYP2D6 genes play a role? A UK population-based study’, *J Psychopharmacol*, vol. 37, no. 4, pp. 396–407, Apr. 2023, doi: 10.1177/02698811231152748.

[2] F. Pishgar, N. Greifer, C. Leyrat, and E. Stuart, ‘MatchThem:: Matching and Weighting after Multiple Imputation’, *The R Journal*, vol. 13, no. 2, pp. 292–305, 2021.

[3] N. Greifer, *cobalt: Covariate Balance Tables and Plots*. (2024). [Online]. Available: https://ngreifer.github.io/cobalt/
